# Whole-genome sequencing reveals three follicular lymphoma subtypes with distinct cell of origin and patient outcomes

**DOI:** 10.1016/j.xcrm.2025.102278

**Published:** 2025-08-07

**Authors:** Weicheng Ren, Mingyu Yang, Xianhuo Wang, Man Nie, Yuhua Huang, Hui Wan, Dongbing Liu, Xiaobo Li, Xiaofei Ye, Bin Meng, Wenqi Jiang, Huiqiang Huang, Zhiming Li, Huilai Zhang, Kui Wu, Qiang Pan-Hammarström

**Affiliations:** 1 Division of Immunology, Department of Medical Biochemistry and Biophysics, Karolinska Institutet, Stockholm, Sweden; 2 BGI Genomics, Shenzhen, China; 3 HIM-BGI Omics Center, Hangzhou Institute of Medicine, Chinese Academy of Sciences, BGI Research, Hangzhou, China; 4 Department of Lymphoma, Tianjin Medical University Cancer Institute and Hospital, National Clinical Research Center of Cancer, Key Laboratory of Cancer Prevention and Therapy, Tianjin, China; 5 Department of Medical Oncology, State Key Laboratory of Oncology in South China, Collaborative Innovation Center for Cancer Medicine, Sun Yat-Sen University Cancer Center, Guangzhou, China; 6 Kindstar Global Precision Medicine Institute, Wuhan, China

**Keywords:** follicular lymphoma, genomics, genetic subtypes, cell of origin, tumor microenvironment, somatic mutations, transcriptomics

## Abstract

Follicular lymphoma (FL) is characterized by clinical, phenotypic, and genetic heterogeneity. Here, we conduct whole-genome sequencing on 131 Chinese FL samples and identify three clinically relevant genetic subtypes. These include C1, associated with favorable prognoses and enriched for *BCL6*-related translocations and mutations in the NOTCH/nuclear factor κB (NF-κB)/immune evasion pathways; C2, characterized by *BCL2-IGH* translocations and mutations in chromatin modifiers; and C3, associated with poorer prognosis, lacking *BCL2-IGH/BCL6*-related translocations but exhibiting more copy number variations. We validate these subtypes in an independent Western cohort (*n* = 227) using the same classification strategy. Transcriptionally, C1 and C3 tumors display signatures of activated B cell-like diffuse large B cell lymphoma (DLBCL), whereas C2 tumors resemble germinal center B cell-like DLBCL. Furthermore, C1 tumors are distinguished from C3 by exhibiting gene signatures of age-associated B cells and an inflamed tumor microenvironment. Our findings illustrate the molecular heterogeneity of FL and define subtypes with distinct cell of origin and clinical outcomes, offering opportunities for personalized therapeutic strategies.

## Highlights

• Whole-genome sequencing defines three genetic subtypes of follicular lymphoma
• Subtypes exhibit distinct mutational and transcriptional patterns and tumor microenvironments
• Subtypes are linked to distinct cell-of-origin characteristics and patient outcomes

## Graphical abstract

**Figure:**
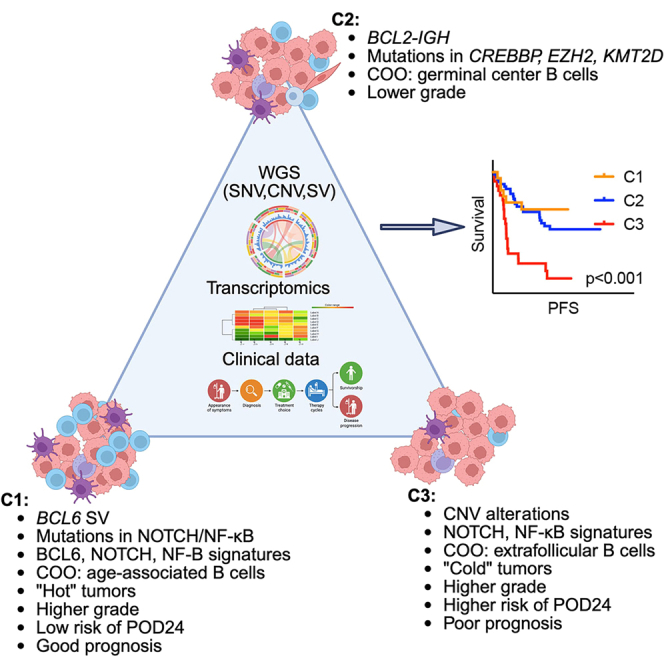

## Teaser

Using whole-genome and transcriptomic sequencing, Ren et al. identify three clinically relevant genetic subtypes of follicular lymphoma. These subtypes are defined by distinct mutation profiles, mutational processes, gene expression programs, tumor microenvironment compositions, clinical outcomes, and cell of origin.

## Introduction

Follicular lymphoma (FL) is a phenotypically, genetically, and clinically heterogeneous disease that accounts for approximately one-third of non-Hodgkin’s lymphoma cases.^1^ FL is a slow-growing lymphoma, but it is currently incurable. While many patients with FL achieve relatively long survival times following various standard therapies, a fraction of patients experience refractory or relapsed (R/R) disease or progress to a more aggressive form of lymphoma called diffuse large B cell lymphoma (DLBCL), which is characterized by a germinal center B cell (GCB)-like phenotype and/or increased interferon regulatory factor 4 (IRF4) expression.^2^^,^^3^ The characteristics of patients with FL, including gender/age at diagnosis, disease stage/grade, survival rates, and incidence, vary significantly across geographic and ethnic groups.^4^^,^^5^^,^^6^ For example, Asian patients with FL have a lower incidence rate,^7^^,^^8^ younger age at diagnosis,^7^^,^^9^ and distinct genetic mutation patterns^10^ compared to Western patients. According to the World Health Organization (WHO) classification, FL includes four major entities: FL, *in situ* follicular B cell neoplasm, pediatric-type FL, and duodenal-type FL.^11^ Recently, the WHO-HERM5 further classified FL into three subentities: classical FL (cFL), follicular large B cell lymphoma (FLBL), and FL with uncommon features (uFL).^12^ cFLs, accounting for 85% of FL cases, are characterized by a follicular growth pattern involving centrocytes and centroblasts and often harbor the t(14;18)(q32;q21) translocation. FLBL, an intermediate subtype between cFL and DLBCL, exhibits either a focal or extensive diffuse growth pattern, whereas uFL represents a new entity comprising two subsets with distinct genetic alterations and outcomes.^12^

Genetically, one of the hallmarks of FL is t(14;18) translocation, which accounts for approximately 80%–90% of cases in Western countries.^2^ This translocation, involving the *IGH* and *BCL2* loci, drives BCL2 overexpression, but alone it is insufficient for lymphomagenesis.^2^ This initiating oncogenic “hit” occurs during VDJ recombination in bone marrow cells, confers resistance to apoptosis, and increases susceptibility to additional genetic alterations in the germinal center (GC), potentially leading to malignancy.^13^^,^^14^ The *BCL2-IGH* translocation is, however, significantly less frequent (47%–60%) in Asian patients with FL,^4^^,^^12^^,^^15^^,^^16^ and its prevalence is even lower among patients with FL with concomitant hepatitis B virus (HBV) infection (8%–33%),^10^^,^^17^ as well as in patients diagnosed with grade 3A/B FL (18%–38%).^15^^,^^16^^,^^18^^,^^19^ Furthermore, a study investigating preneoplastic conditions in healthy individuals in Taiwan reported a *BCL2-IGH* translocation frequency of 10.7%, which was significantly lower than that observed in Western populations (>50%).^20^

Genomic studies have revealed recurrent mutations that contribute to the development of FL, including changes in genes involved in chromatin modification, B cell receptor (BCR), DNA repair, NOTCH, nuclear factor κB (NF-κB), apoptosis, and immune regulation pathways.^10^^,^^21^^,^^22^^,^^23^^,^^24^^,^^25^^,^^26^^,^^27^^,^^28^ These genetic alterations not only contribute to the growth and survival of FL cells but also affect the antitumor immune response, making FL a challenging disease to treat. Efforts have also been made to elucidate the connections between genetic alterations and patient outcomes. Genomic array data have shown that certain copy-number variations (CNVs), such as deletions of 17p, 16p, and 9p, were associated with inferior survival in patients with FL.^29^ The mutation status of seven genes and important clinical parameters were integrated into a clinicopathological risk model (M7-follicular lymphoma international prognostic index [M7-FLIPI]) to identify high-risk patients with short progression-free survival (PFS).^30^ In addition, gene expression and immunohistochemistry-based algorithms have been developed to predict the outcome of patients with FL.^31^^,^^32^ More recently, targeted panel sequencing of single-nucleotide variants (SNVs) has identified several distinct molecular subtypes.^33^^,^^34^ Additionally, a classifier using coding and noncoding mutations has been established to predict the risk of histologic transformation in FL.^35^ However, these molecular subtypes either lack correlations with clinical prognosis or fail to incorporate key FL hallmarks, such as *BCL2-IGH* and *BCL6*-related translocations. Therefore, critical aspects of clinically relevant genetic subtypes and their cell of origin in FL remain insufficiently explored.

Here, we performed whole-genome sequencing (WGS) and transcriptomic sequencing on tumor samples from 131 Chinese patients with FL, identifying three clinically relevant molecular subtypes with distinct mutational patterns/processes, transcriptional profiles, cell-of-origin characteristics, and clinical outcomes.

## Results

### Mutational signatures in FL genomes

**Figure 1:**
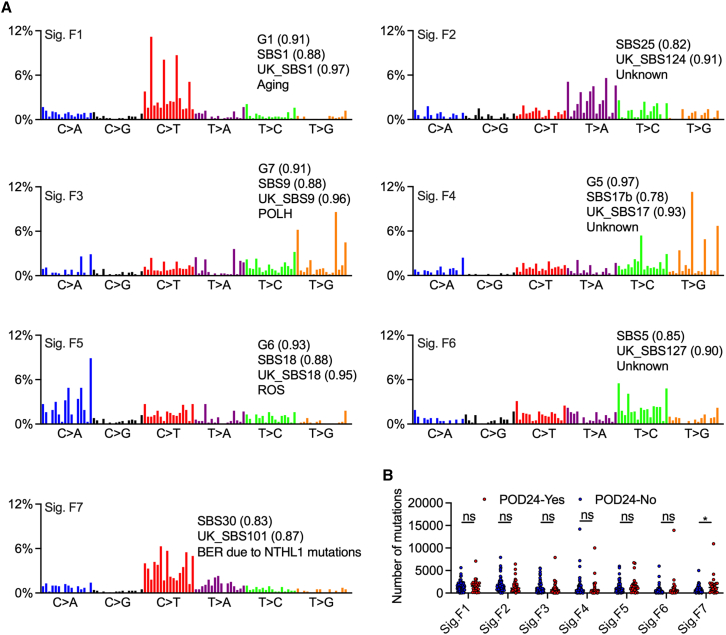
Genome-wide mutational signatures in FLs (A) Mutational signatures were characterized from 131 FL tumor samples sequenced by WGS based on 96 substitution classifications. All identified signatures were compared to those signatures in the indicated databases, and cosine similarity was used to estimate the similarities between the signatures. (B) The mutational signatures of FL tumors with and without POD24 were compared among patients treated with R-CHOP. Data are represented as mean ± SEM. Mann-Whitney U test was used to calculate the *p* value via the nonparametric test. Statistical significance was defined as *p* < 0.05. ∗*p* < 0.05. ns, not significant. Sig, signature; BER, base excision repair; ROS, reactive oxygen species; POLH, DNA polymerase eta; POD24, progression of disease within 24 months. See also Figure S8.

To gain insight into the genetic heterogeneity of FL, we conducted WGS on 131 FL tumors from Chinese patients (Table S1), including 62 tumors with paired non-malignant controls^10^ and 69 tumor-only samples. We first analyzed genome-wide mutational signatures based on 96 mutation types (Figure 1A). Eight robust signatures were identified, including those associated with aging (Sig.F1), DNA polymerase eta (POLH, Sig.F3), reactive oxygen species (ROS, Sig.F5), base excision repair (BER, Sig.F7), and unknown etiology (Sig.F2, Sig.F4, and Sig.F6) (Figure 1A). Sig.F2, resembling SBS25 and UK_SBS124,^36^ has not been reported in a smaller FL cohort.^10^ Notably, SBS25 has been linked to Hodgkin lymphoma and *TNFAIP3* mutations.^37^ Sig.F8, similar to the SBS54 signature in Catalogue Of Somatic Mutations In Cancer (COSMIC), is likely attributed to the germline variants derived from the tumor-only samples. We compared the exposure of Sigs.F1–F7 in R-CHOP-treated patients with FL with and without progression of disease within 24 months (POD24) (Figure 1B). While exposure to Sigs.F1–F6 showed no significant differences between groups, exposure to Sig.F7, associated with BER deficiency due to inactivating mutations in *NTHL1*, was higher in patients with POD24 (*p* = 0.047, Mann-Whitney test). Approximately 11% (*n* = 15) of FL tumors in our cohort carried at least one nonsilent mutation in BER-related genes. Notably, almost all these mutations (94%) were predicted to be damaging *in silico* (by CADD and/or SIFT), indicating potential functional consequences. Compared with tumors lacking nonsilent mutations in BER-related genes, those harboring such mutations presented significantly greater exposure to this signature (median: 1,186 vs. 556; *p* = 0.009, Mann-Whitney test).

**Figure 2:**
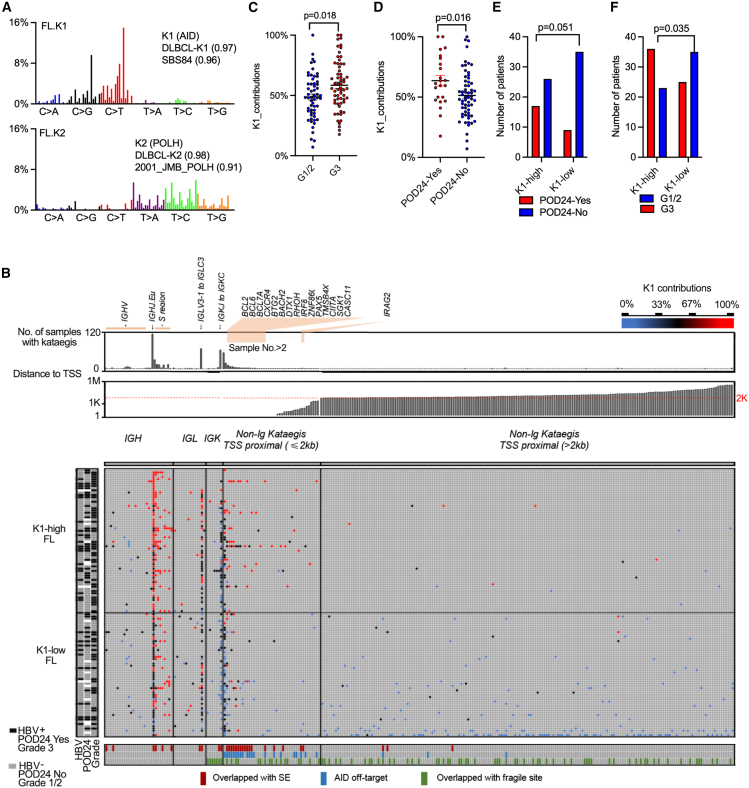
Mutational signatures of clustered mutations (kataegis) in FLs (A) Bar plots showing the two mutational signatures extracted from kataegis in FL genomes. (B) Matrix illustrating the distribution of kataegis regions identified in FL genomes. Each row represents a sample, whereas each column represents the genomic location of the kataegis region. In the matrix, a kataegis is colored based on the contribution of K1, with shading representing the extent of the K1 contribution. The gray bricks indicate the absence of kataegis. The rows were ordered based on the K1 contribution, which was calculated as the average contribution of K1 for all kataegis within each individual sample. The FL tumors were subsequently divided into two groups according to the median K1 contribution as a cutoff: K1-high FL and K1-low FL. The columns were ordered based on genomic location and categorized as Ig loci, non-Ig (TSS proximal) loci, or non-Ig (TSS distal) loci. SE, super-enhancer. (C and D) Dot plots showing the comparison of K1 contributions in the indicated groups. Data are represented as mean ± SEM. Mann-Whitney U test was used to calculate the *p* value via the nonparametric test. Statistical significance was defined as *p* < 0.05. (E) Number of patients with FL with and without POD24 in the indicated groups. In (D) and (E), only patients who received R-CHOP treatment were included. (F) Number of patients with FL with different grades in the indicated groups. (E and F) The χ^2^ test (two-tailed) was used to calculate the *p* value. See also Figure S8.

We subsequently analyzed the mutational signatures of kataegis. Kataegis, first described in breast cancer genomes,^38^ refers to regions of highly clustered mutations within cancer genome and may provide a more precise fingerprint of the underlying mutagenic processes.^39^ We identified 778 kataegis in our cohort, involving 17,707 mutations and constituting approximately 1% of the total number of mutations, with an average of six kataegis per FL genome (Table S1). Two major mutational signatures were extracted from these kataegis, termed Sig.FL-K1 and Sig.FL-K2 (Figure 2A), which are consistent with the previously reported K1 and K2 in DLBCL.^40^^,^^41^ K1 is related to the activation-induced cytidine deaminase (AID) activity and is enriched in activation B cell-like (ABC) DLBCL, whereas K2 is associated with POLH activity and is enriched in GCB-like DLBCL.^40^^,^^41^ The majority of the kataegis events were concentrated in specific genomic regions, notably the *IGH*, *IGK*, and *IGL* genes, along with the transcription start site (TSS)-proximal regions of genes such as *BCL2*, *BCL6*, *BCL7A*, *CXCR4*, and *BTG2* (Figure 2B). To further characterize the association between kataegis and disease progression, we classified kataegis into two categories, K1-dominant kataegis (indicated in red) and K2-dominant kataegis (blue), based on the relative contribution of the K1 and K2 mutational signatures within each kataegis (Figure 2B). K1-dominant kataegis was enriched in the immunoglobulin (Ig) switch (S) regions and TSS-proximal (≤2 kb) regions, whereas K2-dominant kataegis was associated with Ig variable (V) regions as well as TSS-distal regions (>2 kb). We subsequently calculated the average contribution of K1 for all kataegis within each tumor. Notably, we observed a significantly greater contribution of K1 in histologic grade 3 FLs than in grade 1/2 FLs (Figure 2C). Additionally, R-CHOP-treated patients with POD24 presented greater K1 contributions than those without POD24 (Figure 2D), suggesting a potential link between AID-mediated mutational processes and early disease progression in FLs. Furthermore, we divided the FL cases into two groups according to the median cutoff: K1-high FL and K1-low FL (Figure 2B). The K1-high subgroup was significantly associated with an unfavorable prognosis and advanced disease stages, as indicated by a higher incidence of POD24 and a greater incidence of grade 3 tumors (Figures 2E and 2F).

### Significantly mutated genes in coding regions of FL genomes

**Figure 3:**
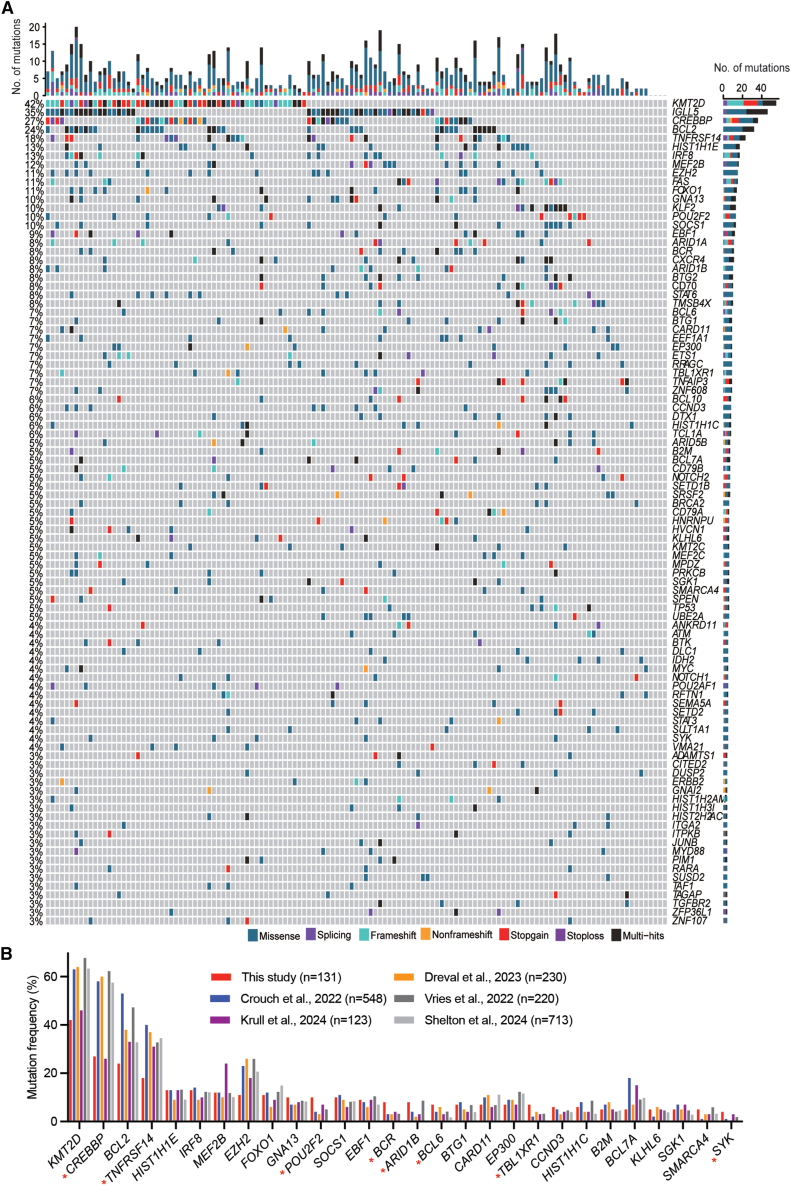
List of significantly mutated genes in the coding regions of FL genomes (A) The genes shown are those affected by somatically occurring nonsilent mutations in FL samples sequenced by WGS (*n* = 131) and are considered significantly mutated (*q* < 0.05; >3%). (B) Comparison of gene mutation frequencies among various cohorts. Only the SMGs in (A) that were found in other cohorts are displayed. *p* values were calculated via the χ^2^ test. Statistical significance was defined as *p* < 0.05. Red ∗ represents the genes with mutation frequencies that are significantly different when our cohort is compared with the other three cohorts. See also Figures S1 and S8.

To identify significantly mutated genes (SMGs) within the coding regions, we applied the IntOGen pipeline and identified a total of 75 SMGs in our cohort (*q* < 0.05), each affected by nonsilent mutations in at least 4 samples (>3%) (Figure 3A). On average, each FL genome harbored seven SMGs, with no notable difference observed between the paired and tumor-only sample cohorts (Table S1). These SMGs affect several well-known factors/pathways involved in lymphomagenesis, including chromatin modifiers (*KMT2D*, *CREBBP*, *HIST1H1E*, *MEF2B*, *POU2F2*, and *EZH2*), transcription factors (*IRF8*, *FOXO1*, *EBF1*, *BCL6*, and *ETS1*), immune modulators (*TNFRSF14*, *CD70*, and *B2M*), factors in the BCR/NF-κB pathway (*CARD11*, *TNFAIP3*, *BCL10*, *CD79A/B*, *BTK*, and *KLHL6*), NOTCH signaling pathway (*KLF2*, *DTX1*, *NOTCH1/2*, and *SPEN*), apoptosis (*BCL2* and *FAS*), and tumor suppression (*TP53*). Notably, genes that are most frequently altered in FL, such as *KMT2D*, *CREBBP*, *BCL2*, *TNFRSF14*, and *EZH2*, exhibited significantly lower mutation frequencies in our cohort than in the five previously reported Western cohorts (Figure 3B).^33^^,^^34^^,^^35^^,^^42^^,^^43^ Conversely, some genes, such as *POU2F2*, *ARID1B*, *TBL1XR1*, *SYK*, *FAS*, *KLF2*, *CXCR4*, *BCL10*, and *CD70*, were more frequently mutated in our cohort (Table S2). The observed discrepancies among the studies may arise from various factors, including differences in ethnic background,^44^ FL grade composition, and the proportion of Hepatitis B surface antigen (HBsAg)-positive cases (Figures S1A–S1C), as well as the inclusion of matched controls and variations in mutation-calling pipelines (Table S2).

### CNV landscapes in FL genomes

**Figure 4:**
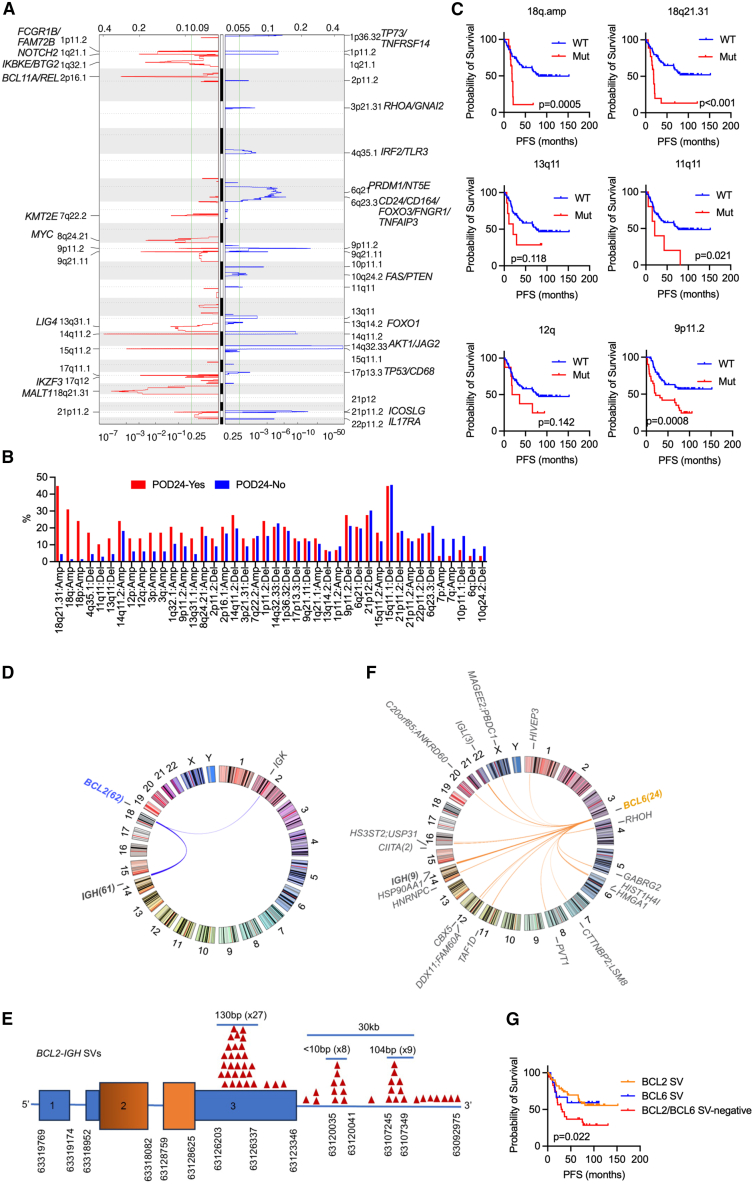
CNV landscape and frequent SVs and their associations with POD24 in FLs (A) The figure illustrates the landscape of CNV alterations in 131 FLs. The amplifications and deletions of CNVs across various chromosomes are depicted. The *y* axis represents the G-score altitude. (B) Bar plots showing the frequencies of the indicated CNV alterations in FLs with and without POD24. Only patients treated with R-CHOP were included in the analysis. (C) Kaplan-Meier survival analysis illustrating progression-free survival (PFS) in FLs with and without the indicated CNV alterations among patients who received R-CHOP treatment. (D–G) Analysis of *BCL2* and *BCL6* SVs identified in 131 FL genomes. (D, F) The figure illustrates the identified SVs involving *BCL2* (D) and *BCL6* (F) in FLs. (E) The diagram displays the SVs identified in the *BCL2* locus. Translocation breakpoints involving *BCL2* are indicated by red triangles. (G) Kaplan-Meier survival analysis illustrating PFS in the indicated groups among patients who received R-CHOP treatment. (C and G) The *p* value was calculated via the log rank test. See also Figure S8.

To understand the genome-wide CNV patterns in FLs, we analyzed recurrent CNVs in our WGS cohort using GATK and GISTIC2.0 pipelines^45^ and validated the results using FACETS.^46^ This analysis revealed a significant ensemble of 640 focal CNV regions, including 243 gains and 397 losses (Figure 4A). On average, each genome harbored five CNVs (range: 0–14), comprising two gains and three losses per genome, with individual CNV frequencies ranging from 2% to 26%. The most prevalent CNVs included deletions at 21p12/*BAG* (26%), 14q32.33 (24%), and 9p11.2 (21%) and amplifications at 14q11.2 (22%), 18q21.31/*MALT1* (18%), 2p16.1 (18%), and 8q24.21/*MYC/PVT1* (16%) (Figure 4A). Notably, gains at 18q21.31, 8q24.21, and 14q11.2 and losses at 4q35.2, 11q11, and 13q11 were enriched in R-CHOP-treated patients with POD24 (Figure 4B). Among these, gain of 18q21.31 and loss of 11q11/9p11.2 were closely associated with inferior PFS in patients treated with R-CHOP, indicating their potential as predictive markers (Figure 4C).

### *BCL2* and *BCL6* translocations in FLs

We next characterized recurrent interchromosomal structural variants (SVs) in FLs using Manta^47^ and further identified *IGH*-related SVs using SeekSV.^48^ This analysis revealed *BCL2*, *BCL6*, and *IGH* as the most frequently rearranged genes (Figure 4D). *BCL2-IGH* translocations were present in approximately 47% of the patients in our cohort, which is consistent with the findings of a previous study indicating a relatively lower occurrence of *BCL2-IGH* translocations in Chinese patients with FL.^15^ At the *BCL2* locus, we assessed the sequencing performance and observed a median sequencing depth of 50× (26×–243×), with 10× and 20× coverage at 99.4% and 98.6%, respectively. Importantly, these metrics were comparable between FLs with and without *BCL2-IGH* translocations (Figures S1D–S1G), suggesting that sequencing performance at the *BCL2* region was sufficient to detect translocations. This makes it unlikely that technical limitations contributed to the low frequency of *BCL2-IGH* translocations observed in our cohort. Moreover, all *IGH* breakpoints occurred within *V(D)J* regions, which is consistent with the hypothesis that these events may occur during *V(D)J* recombination in pre-B cells.^49^ Notably, most breakpoints within *BCL2* predominantly clustered in the 3′ untranslated region (UTR) and downstream regions, 77% of which were detected in three hotspots: a 130-bp segment (*n* = 27) in the 3′ UTR, a 10-bp segment (*n* = 8) ∼3.3 kb downstream, and a 104-bp segment (*n* = 9) ∼16 kb downstream (Figure 4E).

Translocations involving *BCL6* were identified in 18% of our cohort, consistent with previous reports (13%–24%) based on fluorescence *in situ* hybridization or targeted sequencing.^15^^,^^42^^,^^50^^,^^51^^,^^52^ In addition to its primary partner *IGH*, *BCL6* translocation involves other partner genes, such as *IGL*, *HNRNPC*, *HMGA1*, *PVT1*, *HIST1H4I*, *GABRG2*, *CIITA*, *LSAMP*, and *RPSA*, highlighting a network of diverse fusion events involving the *BCL6* locus in FLs (Figure 4F). Notably, all *IGH* breakpoints involved in *BCL6-IGH* translocations occur in the S regions, which consist of repetitive sequences that mediate Ig class switch recombination,^53^ suggesting that these translocations arise through a mechanism distinct from *BCL2-IGH* translocations. Interestingly, R-CHOP-treated patients with FL lacking *BCL2-* and *BCL6*-related translocations presented poorer prognostic outcomes than those who harbored these translocations (Figure 4G).

### Genetic alterations and transcriptomic phenotypes capture the disease heterogeneity of FL

**Figure 5:**
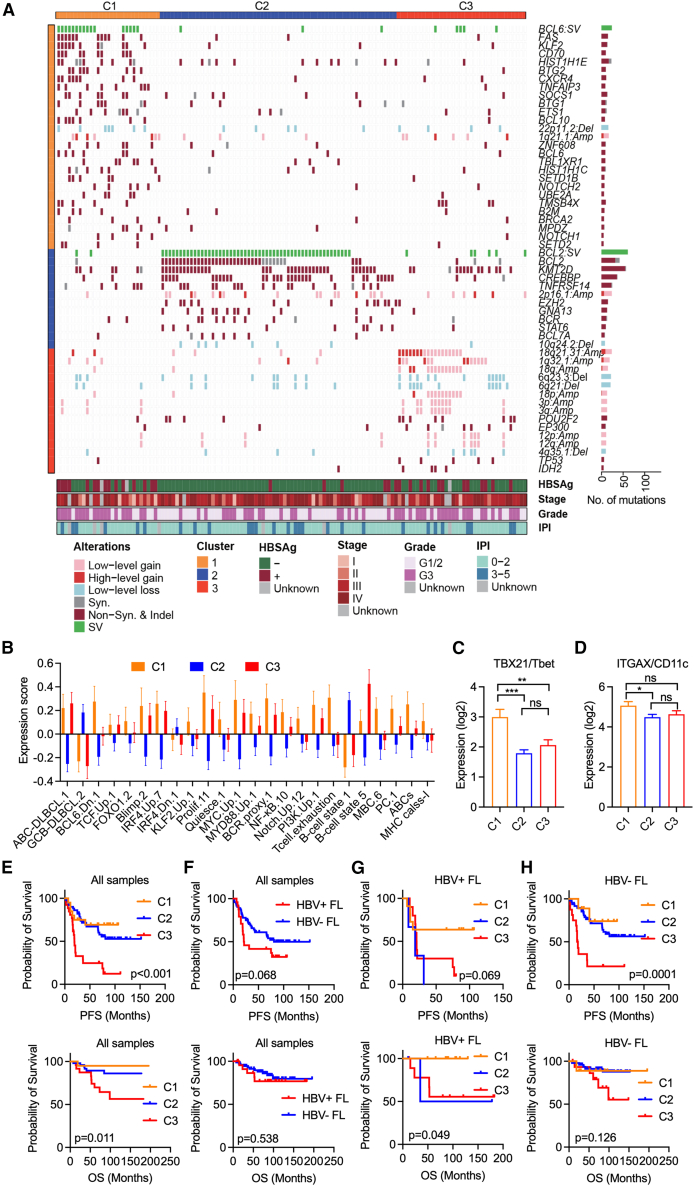
Identification of FL molecular subtypes with distinct genetic mutation patterns and transcriptional phenotypes (A) By combining SMGs, CNVs, and SVs, a nonnegative matrix factorization consensus clustering approach was used to classify 131 FL tumors. Clusters C1–C3 are depicted with their respective markers highlighted within boxed regions for each cluster. *p* < 0.05 was used to define the markers of individual clusters. Fisher’s exact test was used to calculate the *p* value. (B) Comparison of the expression signatures of the indicated pathways among different FL subtypes. The expression signatures were identified via gene sets available at https://lymphochip.nih.gov/signaturedb/, employing established methodologies as described in a previous study.^54^ The gene set used for the age-associated B cell signature is described in Figure 1 of a previous study.^55^ (C and D) Expression of the indicated genes among various clusters. Data are represented as mean ± SEM. The Mann-Whitney U test was used to calculate the *p* value via the nonparametric test. Statistical significance was defined as *p* < 0.05. ∗*p* < 0.05, ∗∗*p* < 0.01, ∗∗∗*p* < 0.001. ns, not significant. (E–H) Kaplan-Meier survival analysis illustrating the differences in PFS and overall survival (OS) among different FL subtypes in the indicated groups among patients who received R-CHOP treatment. The *p* value was calculated via the log rank test. MBCs, memory B cells; PCs, plasma cells; Dn, downregulated; Prolif, proliferation; Syn, synonymous; Nonsyn, nonsynonymous; G, grade. See also Figures S2–S5.

FL tumors harbor variable numbers of genetic drivers, and individual mutations may influence their development, progression, and prognosis. Understanding co-occurring genomic modifications could provide deeper insights into the heterogeneity of the disease. Combining SMGs, CNVs, and hallmark interchromosomal SVs (*n* = 127 markers; Table S3), we applied nonnegative matrix factorization consensus clustering to elucidate the spectrum of these genetic alterations in our FL cohort. This approach identified three robust FL clusters, each defined by distinct genomic features (Figures 5A, S2A, and S2B). Furthermore, transcriptomic analysis (*n* = 104) revealed gene expression signatures specific to each cluster (Figures 5B and S2C–S2E).

Cluster C1, comprising 29 FLs, was characterized by prevalent *BCL6* SVs (>55%) and co-occurring mutations in genes in key signaling pathways, including NOTCH (*NOTCH1/2* and *KLF2*), BCR/NF-κB (*TNFAIP3*, *BCL10*, and *TBL1XR1*), immune evasion (*CD70* and *B2M*), and apoptosis/cell cycle regulation (*FAS* and *BTG1/2*) (Figure 5A). This genetic profile, lacking *BCL2-IGH* SVs and chromatin modifier mutations, resembled that of C1/BN2-DLBCL^56^^,^^57^ and certain types of marginal zone lymphomas (MZLs).^58^ Consistent with these alterations, transcriptomic analysis revealed enriched signatures associated with BCL6-, KLF2-, and NOTCH-target genes, ABC-DLBCL, apoptosis, BCR/NF-κB, and T cell exhaustion, along with elevated expression of individual genes including *IRF4*, *NFKBIE*, *PIM1*, *BTG2 ETV6*, and *BLNK* (Figures 5B and S2C). Notably, C1 tumors not only presented a gene signature associated with age-associated B cells but also showed increased expression of canonical markers of these cells, such as *TBX21*/Tbet and *ITGAX*/CD11c (Figures 5C and 5D), indicating a transcriptomic phenotype resembling that of age-associated B cells. Furthermore, these tumors exhibited GC-derived memory B cell characteristics^59^ and elevated expression of major histocompatibility complex (MHC)-I molecules (Figure 5B), features commonly observed in age- or autoimmune-associated B cells.^55^^,^^60^

Cluster C2, comprising 66 FLs, exhibited a conventional FL genetic profile, characterized by a high frequency of *BCL2-IGH* translocations (>82%) and mutations in chromatin modifiers such as *KMT2D*, *CREBBP*, and *EZH2* (Figure 5A). Additional recurrent mutations were detected in genes such as *BCL2*, *GNA13*, *TNFRSF14*, *BCL7A*, and *STAT6*, which are commonly altered in conventional FLs and GCB-DLBCL. Transcriptomic analysis revealed elevated expression of *MME*, *S1PR2*, and *BACH2* (Figure S2C), along with distinct expression patterns characterized by a strong GCB-DLBCL signature (Figures 5B and S2E). Furthermore, these tumors exhibited a robust stromal signature and a GC B cell signature (B cell state_S1) (Figure 5B), both of which have been associated with a favorable prognosis in patients with DLBCL.^61^^,^^62^ These findings support the classification of C2 as representative of conventional FL.

Cluster C3, comprising 36 FLs, lacked both *BCL6* and *BCL2-IGH* SVs but presented recurrent mutations in *TP53*, *POU2F2*, *IDH2*, and *EP300*, along with multiple CNVs (Figure 5A). Notably, CNV gains in 18q21.31/*MALT1* and 1q32.1/*PIK3C2B/BTG2/KISS1* and CNV losses in 6q23.3/*TNFAIP3*, 6q21/*NT5E/PRDM1/BACH2* were more frequent in this cluster, potentially affecting phosphatidylinositol 3-kinase (PI3K) and NF-κB signaling and pathways associated with plasma cell differentiation (Figure 5B). Despite comparable tumor contents across clusters, C3 tumors harbored significantly more CNV drivers than C1/C2 tumors (Figures S2F and S2G). Transcriptionally, C3 tumors resembled those of C1 tumors (Figures S2A and S2B), with enriched expression of signatures associated with ABC-DLBCL, proliferation, BCR/NF-κB, and PI3K pathways (Figure 5B). Moreover, C3 tumors exhibited a B cell state_S5 signature (Figure 5B), characteristic of preplasmablasts and associated with ABC-DLBCL and poor prognosis in patients with DLBCL.^62^

### Associations of clinical characteristics and outcomes with genetic clusters in patients with FL

We next assessed the clinical relevance of the three genetic clusters (Figures 5E–5H; Table S4). First, scores generated from models such as FLIPI, m7-FLIPI, and PRIMA-prognostic index (PRIMA-PI)^63^ did not correlate with the genetic profiles of each cluster. Second, high-grade (G3) tumors were more common in the C1 (76%) and C3 subtypes (64%) than in the C2 subtype (35%) (*p* < 0.01, Fisher’s exact test). Third, immunohistochemistry staining revealed that C1 and C3 tumors had a lower proportion of CD10-positive tumors (71% and 71%, respectively, vs. 98%) and a higher proportion of multiple myeloma oncogene 1 (MUM1)-positive tumors (36% and 52%, respectively, vs. 20%) than C2 tumors, whereas BCL2 (>93%) and BCL6 (>89%) positivity was consistent across all clusters. Fourth, concomitant HBV infection was significantly more prevalent among patients in the C1 (56%) and C3 (48%) subtypes than those in the C2 subtype (11%). Fifth, among R-CHOP-treated patients (*n* = 97), POD24 was more frequently observed in the C3 subtype (68%) than in the C1 (25%) or C2 (14%) subtypes (Table S4). This finding was further supported by the observation that C3 subtype had less favorable outcomes, including significantly shorter PFS and overall survival (Figure 5E). Moreover, although HBV-positive patients were associated with poorer prognoses, especially those in the C2 and C3 subtypes (Figures 5F–5H), excluding them from the analysis did not significantly alter the genetic/clinical features of each subtype (Figures 5H and S3A; Table S5). Furthermore, multivariable Cox analysis, including age, international prognostic index (IPI), stage, grade, and M7-FLIPI, confirmed that the genetic subtypes independently predicted treatment outcomes in R-CHOP-treated patients (*p* = 0.007; Figure S3B).

Next, we attempted to incorporate additional genetic features, including kataegis signatures (K1/K2, Figure 2B) and noncoding drivers (Table S3), into our clustering approach. Nonetheless, ∼90% of patients remained in their original clusters, with a slight improvement in prognostic stratification when all features were considered (Figures S3C and S3D). Additionally, we applied recently published FL subtyping methods to our cohort and reproduced similar clusters^32^^,^^33^^,^^34^^,^^35^; however, PFS differences across these clusters were not significant (Figures S4A–S4K). Notably, 86% of the C1 tumors were classified as DLBCL-like FL (dFL), which was higher than that of C2 (60%) and C3 (53%) tumors (Figure S4A), suggesting a higher risk of histologic transformation of C1 tumors. Furthermore, using the previously described Bernoulli mixture model^33^^,^^34^ with the markers characterized in our cohort, we identified four clusters that closely resembled the original three (Figures S4L–S4O), with Akaike information criterion (AIC)-C4 (C3-like) showing the poorest PFS. These results highlight the importance of integrating CNVs and key SVs with SNVs to better define clinically relevant subtypes.

### Validation of FL genetic clusters in an independent WGS cohort

To validate the genetic clusters, we applied the same approach to an independent cohort of 227 FL tumors with available SNV, SV, and CNV data.^35^ This analysis identified three clusters that recapitulated the key genetic features observed in our classification (Figure S5A). Specifically, C1 showed frequent *BCL6* translocations, 1q21.1_amplification, and *SOCS1*, *CD70*, and *NOTCH1* mutations; C2 was enriched for *BCL2-IGH* translocations and *CREBBP*, *KMT2D*, *BCL2*, *TNFRSF14*, and *EZH2* mutations; and C3 was again characterized by prevalent CNVs. Although certain markers showed less overlap between cohorts, C3-associated CNVs remained more enriched in C3 tumors than in C1/C2 tumors in our cohort, and the original C1-defining mutations retained higher frequencies in C1 tumors from the validation cohort (Figures S5B and S5C). Furthermore, clustering analysis of the combined cohorts (*n* = 358) identified three consistent clusters (Figure S5D), supporting the stability and reproducibility of these genetic subtypes across different patient populations. In the validation cohort, C1 and C3 tumors were more frequently associated with high grade (G3) (Figure S5E), and notably, 64% of C3 patients failed to achieve complete remission following R-CHOP treatment (Figure S5E), indicating poorer prognoses. These correlations further validated the clinical relevance of the observed genetic clusters.

### Distinct mutagenesis processes in various FL genetic subtypes

**Figure 6:**
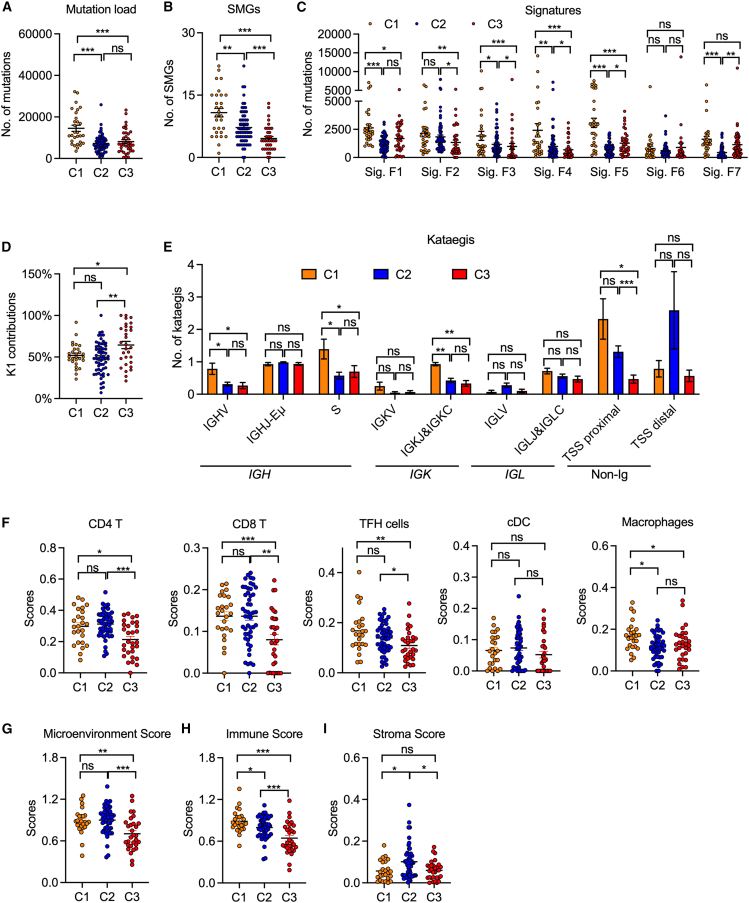
Distinct mutagenesis processes and composition of the TME among different FL subtypes (A and B) Dot plots showing the comparison of total mutation load (A) and SMGs (B) among FL subtypes. The total mutation loads were calculated by using the total number of mutations from Sigs.F1–F7. (C) Dot plots showing the comparison of the exposure of the indicated signatures across different FL subtypes. (D) Dot plots showing the K1 contribution among different FL subtypes. (E) Bar plots showing comparisons of the number of kataegis events identified from different genomic locations among different FL subtypes. (F–I) RNA-seq data were used to predict tumor-infiltrating immune cells via the online tool xCell. T follicular helper (TFH) was predicted using CIBERSORTx,^64^ due to its lack in xCell tool. (F) Dot plots showing the comparison of different types of tumor-infiltrating immune cells among FL subtypes. (G) Dot plots showing various scores associated with the TME (G) and immune (H) and stromal (I) signatures among different FL subtypes. Data are represented as mean ± SEM. The Mann-Whitney U test was used to calculate the *p* value via the nonparametric test. Statistical significance was defined as *p* < 0.05. ∗*p* < 0.05, ∗∗*p* < 0.01, ∗∗∗*p* < 0.001. ns, not significant. See also Figure S6.

Next, we investigated the mutagenic context of each genetic cluster by examining the prevalence of SMGs and the individual mutational signatures depicted in Figure 1. Among clusters, the total mutation load, calculated based on mutations attributed to Sigs.F1–F7 (excluding germline-associated Sig.F8), was significantly higher in C1 tumors than in C2 and C3 tumors (Figure 6A). Accordingly, C1 presented the highest number of SMGs, whereas C3 presented the lowest (Figure 6B). Moreover, compared with C2 tumors, C1 tumors presented the greatest exposure to Sigs.F1–F5, whereas C3 tumors presented minimal exposure to Sigs.F2–F4 but higher Sig.F5 and Sig.F7 (Figure 6C). Notably, the exposure of Sig.F3, which resembles the previously reported signature G7 that correlates with the activity of POLH and the rate of somatic hypermutation (SHM) in *IGHV* regions in B cell lymphomas,^40^^,^^41^ was significantly elevated in C1 and C2 tumors compared with C3, with C1 also showing the greatest exposure. We then extended our analysis to compare the kataegis signatures in the different clusters (Figures 6D and 6E). The K1 (AID) and K2 (POLH) signatures contributed similarly to C1 and C2, whereas C3 tumors were predominantly associated with the K1 signature (Figure 6D). Compared with C2 and C3 tumors, C1 showed the highest frequency of kataegis events, particularly in the *IGHV*, *S*, and *IGKJ&C* regions. In the non-Ig regions, C3 tumors had fewer kataegis, especially in the TSS-proximal regions (Figure 6E). Notably, kataegis in C1 tumors was distributed across multiple genes, including *BCL6*, *BCL7A*, *CXCR4*, *BTG2*, *BACH2*, and *DTX1*, whereas in C2 tumors, ∼80% of kataegis events localized to the *BCL2* locus. Consistent with this, C1 tumors had a higher number of mutations in previously defined aberrant SHM (aSHM) regions,^35^ such as *BCL6_TSS*, *DTX1_TSS*, *RHOH_TSS*, *BCL7A_TSS*, and *CXCR4_TSS*, whereas C2 mutations were identified primarily at *BCL2_TSS* (Figure S6A). These findings suggest that mutagenesis processes may vary among different genetic clusters. Mutagenesis in C1 and C2 was driven by both AID and POLH, with C1 tumors exhibiting a higher overall mutation load and a greater number of kataegis in the Ig locus and aSHM regions, suggesting a history of stronger antigen stimulation and/or GC reactions. In contrast, the mutagenesis of C3 tumors appears to be predominantly influenced by AID activity, suggesting a distinct extrafollicular origin for this group.

### Diverse *IGHV* repertoires in different genetic subtypes

We next analyzed *IGHV* gene usage in our cohort and found that 17% (13/76) of tumors expressed the *VH4-34* gene (Figure S6B), which is rarely used in normal B cells and has been linked to autoimmune diseases.^65^^,^^66^ C1 tumors frequently expressed *VH4-34* (18%), *VH1-81* (18%), and *VH3-33* (12%) genes, whereas C3 tumors predominantly expressed *VH4-34* (25%) and *VH3-23* (21%) genes. Consistently, both C1 and C3 tumors exhibited upregulated signaling pathways associated with systemic lupus erythematosus in the Kyoto Encyclopedia of Genes and Genomes database (Figure S6C). FL is thought to originate from dysregulated GC B cells, which would suggest that tumor cells have undergone SHM. Accordingly, we examined SHM in the *IGHV* and observed a mean rate of 7.8% (Figure S6D), which was slightly lower than that in GCB-DLBCL (9.8%) and ABC-DLBCL (8.7%). Notably, C1 and C3 tumors presented similarly low SHM rates (6.8%), which were significantly lower than that of C2 tumors (9.5%) (Figure S6E). Additionally, IgM expression was detected in 62% of the FLs, with a higher prevalence in C1 (75%) and C3 (79%) tumors than in C2 tumors (42%) (Figure S6F). This pattern mirrors the SHM rate and isotype usage observed in DLBCL subtypes, with ABC-DLBCL tumors exhibiting a lower SHM rate and higher IgM usage than GCB-DLBCL tumors do.^67^ Notably, over 86% of the *VH4-34* segments were paired with IgM and exhibited lower SHM than the other *IGHV* (Figures S6G and S6H). These findings suggest that C2 tumors likely originate from isotype-switched B cells with higher levels of SHM, whereas C1 and C3 tumors may arise from unswitched, autoreactive B cells characterized by lower SHM.

### Distinct tumor microenvironment in FL genetic subtypes

To explore the tumor microenvironment (TME) features of FLs within different clusters, we used an analytical tool to predict the abundance of various immune cell types via RNA sequencing (RNA-seq) data.^68^ We assessed the associations between the predicted immune cell scores and patient outcomes in patients with FL treated with R-CHOP. Higher immune and microenvironment scores were associated with better patient outcomes, findings that can be validated in an independent online cohort (GEO: GSE119214, *n* = 137) (Figure S7A). Further analysis of our cohort revealed that C3 tumors presented the lowest levels of infiltration of CD4^+^ and CD8^+^ T cells (Figures 6F and S7B), as did T follicular helper cells, whereas C1 tumors presented significantly increased infiltration of these cells (Figure 6F). Moreover, when the overall TME feature was assessed, C1 tumors presented the highest microenvironment and immune scores among the different genetic clusters (Figures 6G and 6H), suggesting a more inflamed and active microenvironment in these tumors. Notably, the infiltration of macrophages, dendritic, and stromal cells did not differ substantially among the clusters (Figures 6F and 6I), although some cell types may be underrepresented due to limitations in tissue processing.^69^ Overall, these results highlight that FL genetic subtypes were associated with distinct TME compositions: C3 tumors, which were linked to poor prognosis, exhibited a less inflamed TME, whereas C1 tumors showed a more immune cell-infiltrated and potentially immunoresponsive microenvironment.

## Discussion

**Figure 7:**
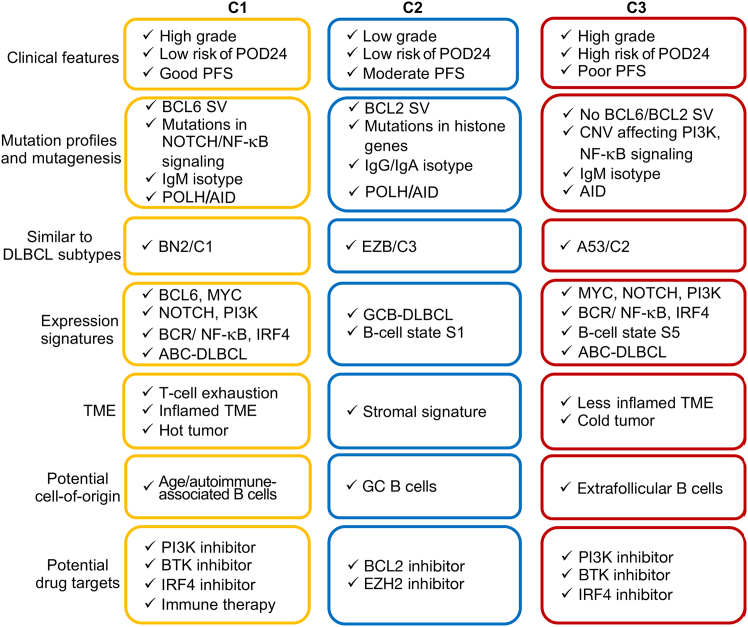
Implications of the FL molecular subtypes for pathogenesis and targeted therapies This figure summarizes the clinical associations, key genetic alterations, gene expression signatures, and potential treatment implications for each FL molecular subtype. The DLBCL genetic subtypes used were described in previous publications.^56^^,^^70^ SV, structural variant; TME, tumor microenvironment; PFS, progression-free survival; POD24, progression of disease within 24 months. See also Figure S7.

Genetic classification has become increasingly important for understanding the biology and heterogeneity of lymphoma, predicting disease outcomes, and guiding clinical trial designs and treatment decisions. In our study, by integrating genomic drivers derived from various types of alterations, we identified three clinically relevant FL genetic subtypes. These subtypes display distinct mutation profiles, mutagenesis patterns, transcriptomic features, compositions of the TME, clinical manifestations, and patient outcomes (Figure 7). Furthermore, our data suggest a potentially distinct cell of origin associated with each subtype: C1, age-associated B cell, atypical memory B cell-like; C2, GC B cell-like; and C3, potentially preplasmablast derived via the extrafollicular B cell pathway. These findings demonstrate the genetic heterogeneity of FL, highlighting the potential for developing therapies tailored to unique molecular profiles (Figure 7).

Unlike DLBCL, whose genetic subtypes have been extensively studied, the genetic classification of FL remains less well defined. Although recent studies have identified distinct genetic clusters via SNVs,^33^^,^^34^ these analyses were limited by the use of targeted sequencing panels, which fail to capture other critical types of genetic alterations, such as CNVs and SVs. WGS allows us to explore different types of genetic alterations comprehensively and identify various molecular subtypes of FLs, especially those with a high risk of early disease progression. Moreover, our analysis further suggested that these molecular subtypes had distinct developmental paths. C2, characterized by *BCL2-IGH* translocations and mutations in chromatin modifiers, represents conventional FL cases with typical follicular growth patterns, encompassing the majority of classic FLs in the latest WHO-HERM5 classification.^12^ This subtype may arise from developmental blockade in the GC, as evidenced by the presence of typical genetic hallmarks of FL and transcriptional phenotypes resembling GCB-DLBCL^56^^,^^70^^,^^71^ and transformed FL.^23^ This is also supported by the relatively high SHM rate and frequently switched Ig isotypes observed in this subtype, suggesting a common origin with GCB-DLBCL.^30^ Further analysis of the C2 cluster via various clustering strategies did not reveal subclusters significantly associated with clinical outcomes. However, certain genetic alterations within the C2 cluster may be associated with poor prognosis. Additionally, immune-related expression signatures reflecting the TME or single-cell RNA-seq may provide further features for subgrouping these tumors.^32^^,^^69^

The C1 and C3 subtypes represent *BCL2-IGH*-negative FLs, each exhibiting distinct genetic mutation patterns, gene expression profiles, and clinical outcomes, which is consistent with previous findings that *BCL2-IGH*-negative FLs exhibit considerable genetic, transcriptional, and clinical diversities.^72^^,^^73^ The C1 subtype, associated with favorable prognosis, includes tumors harboring *BCL6*-related translocations and mutations in genes such as *KLF2*, *CD70, HIST1H1E*, *TNFAIP3*, *ETS1*, *NOTCH1/2*, and *BCL10*. This mutational profile resembles that of C1/BN2-DLBCL^56^^,^^57^ and certain types of MZLs. Additionally, elevated AID/POLH-mediated mutagenesis and *BCL6-IGH* breakpoints in the *IGH S* region suggest that tumor cells may have undergone GC reactions. This subtype also exhibits increased usage of autoreactive *VH4-34* and IgM isotypes and displays a transcriptomic phenotype resembling age-associated B cells with characteristics of memory B cells and higher MHC class-I expression. This mirrors the preferential usage of *VH4-34* observed in nodal MZLs^74^^,^^75^ and in FLs without *BCL2-IGH* translocations.^76^ Together, these findings support the notion that age-associated B cell-like cells, sometimes referred to as atypical memory B cells, particularly autoreactive unswitched clones, may be involved in lymphomagenesis in C1 tumors. This finding is reminiscent of the association between aged/autoimmune B cells and *MYD88*-mutated extranodal DLBCL.^77^ However, C1 tumors appear distinct from recently described memory-like FL subtypes based on gene expression profiles,^32^ as we did not observe their enrichment within our genetically defined clusters. Furthermore, C1 tumors exhibit increased infiltration by various T cell subtypes and an exhausted phenotype within the TME. Previous studies have shown that FLs with *BCL6* translocations and a “hot” TME correlate with improved patient outcomes,^78^^,^^79^ implying a potential connection between the dysregulation of BCL6 and enhanced immune cell recruitment to the TME in C1 tumors. Conversely, the C3 subtype, associated with poorer prognoses, includes tumors predominantly harboring CNV alterations, mirroring the genetic pattern observed in the A53-DLBCL subtype with poor prognoses.^57^ This subtype also displays enriched signatures of ABC-DLBCL, preplasmablasts (distinct B cell state), and “cold” TMEs, as well as a dominant AID-driven mutagenesis, a feature of ABC-DLBCL^41^ and highly proliferating extrafollicular B cells.^80^ These findings suggest that C3 tumors may originate from extrafollicular B cell-like cells,^81^ similar to the cell of origin of subgroup of ABC-DLBCL.^41^ Furthermore, similar to C1 tumors, C3 tumors preferentially use autoreactive *VH4-34* and IgM isotypes and showed enrichment in autoimmune-related signaling pathways,^76^ suggesting that the BCRs expressed by these tumors may recognize self-antigens, potentially promoting malignant transformation or tumor growth.^67^ Overall, this FL classification highlights the complexity of lymphomagenesis and holds promise for targeted therapeutic strategies. For example, C1 tumors may respond to immune checkpoint blockade therapies, whereas C1 and C3 patients may benefit from PI3K inhibitors,^82^ IRF4 inhibitors,^83^ or Bruton's tyrosine kinase (BTK) inhibitors.^84^ C2 patients, on the other hand, may benefit from BCL2 and EZH2 inhibitors (Figure 7).

In our study, we revealed various mutational processes that may drive the development of FLs. Our analysis suggested a link between AID-driven mutagenesis and early disease progression (POD24). Notably, the C3 subtype, which is associated with poor prognosis, exhibited greater AID-driven mutational activity than the C1 and C2 subtypes did (Figure 6D). This finding mirrors observations in DLBCL, where the ABC subtype, compared to the GCB subtype, displayed greater AID-driven mutational activity and was associated with poor prognosis.^41^ Using data derived from whole-exome sequencing (WES) or targeted sequencing, other studies also identified FL subtypes associated with AID signatures,^33^^,^^43^ although they did not establish a link between this process and clinical outcomes in their cohorts. Additional mutational signatures, including those related to POLH (Sig.F3) and ROS (Sig.F5), may shape the mutational landscape of FL subtypes. Thus, in addition to improving our understanding of lymphomagenesis, some of these mutational signatures may also serve as biomarkers for prognostic assessment.

Previous studies, including ours, have shown that tumors from patients with FL with HBV seropositivity exhibit distinct mutation patterns, gene expression profiles, and clinical outcomes.^10^^,^^17^ Our analysis revealed that these HBV-positive FLs were predominantly classified into C1 and C3 subtypes, which may explain the differences observed between Chinese and Western cohorts, such as the lower prevalence of *BCL2-IGH* translocations and the lower mutation frequency of the epigenetic modifiers (*KMT2D*, *CREBBP*, and *EZH2*) in Chinese patients (Figures 3B and S1A; Table S2). While these differences could confound genetic clustering, excluding HBV-positive tumors from the analysis revealed that the genetic and clinical features of the subtypes remained consistent, suggesting minimal impact from including HBV-associated samples in our cohort. Furthermore, although variations in mutation patterns have been observed across various FL cohorts with different ethnic backgrounds, grade compositions, HBsAg-positive cases, and mutation-calling pipelines,^7^^,^^10^^,^^17^^,^^35^^,^^85^ our subtyping approach successfully categorized FL tumors from Western populations into three largely similar FL genetic clusters.^35^ This consistency supports the robustness of our subtypes across populations.

Our study provides a comprehensive WGS-based characterization of FL, identifying three genetically and clinically relevant molecular subtypes with different developmental origins. These findings are important, as current diagnostic methods—relying on morphology and immunohistochemistry—are insufficient to distinguish the C1 and C3 subtypes from the conventional C2 subtype. While ∼80% of Western patients with FL fall into the C2 cluster, identification of the smaller C1 and C3 clusters remains crucial, as they may require different treatments. This classification could be particularly beneficial for patients in HBV-endemic or ethnically diverse regions (South/East Asia, Latin America, and Africa). Integrating this classification into clinical practice and trial design could enhance precision treatment and improve patient outcomes.

### Limitations of the study

Several limitations should be acknowledged. First, although our cohort reflects the clinical heterogeneity of real-world Asian FL populations, potential biases related to distinct demographic or clinical features, such as ethnic origin and different environmental exposures, cannot be excluded. Second, while our analytical strategies indicated that the absence of paired normal DNA had minimal impact on the identification of key somatic events, the influence of residual germline variants—particularly in noncoding regions—cannot be fully ruled out. Third, the TME analysis was primarily based on deconvolution of bulk RNA-seq data and would benefit from further validation using single-cell RNA-seq. Fourth, we proposed distinct developmental trajectories and cells of origin for different subtypes based on the interpretations of their characteristics; further studies are needed to confirm their exact origins. Lastly, although the clustering framework was reproducible in the Western cohort, comprehensive validation of mutation profiles was limited. Moving forward, achieving international consensus on the molecular subtyping of FL will require a collaborative, multi-cohort, and data-driven effort. As demonstrated in DLBCL, where competing classification schemes eventually converged into integrated genomic models (LymphGen, DLBclass, and HMRN), future progress in FL will depend on harmonizing genomic, transcriptomic, and clinical data. Therefore, prospective and larger multi-cohort studies are necessary to confirm the clinical and prognostic relevance of the proposed FL subtypes.

## Resource availability

### Lead contact

Further information and requests for resources and reagents should be directed to and will be fulfilled by the lead contact, Qiang Pan-Hammarström (qiang.pan-hammarstrom@ki.se).

### Materials availability

This study did not generate new unique reagents.

### Data and code availability

• The sequencing data have been deposited in the China National Genebank (https://db.cngb.org/) with accession number CNP0005740.
• This article does not report original code.
• Any additional information required to reanalyze the data reported in this article is available from the lead contact upon request.

## Acknowledgments

This work was supported by the 10.13039/501100002794Swedish Cancer Society, the 10.13039/501100004359Swedish Research Council, Radiumhemmets, the Mayo-KI collaborative grant, the 10.13039/501100004063Knut and Alice Wallenberg Foundation, the O.E. and Edla Johansson Foundation and the Guangdong Provincial Key Laboratory of Human Disease Genomics (2020B1212070028), 10.13039/501100001809National Natural Science Foundation of China (W2412122), and China National GeneBank (CNGB).

## Author contributions

W.R. and M.Y. analyzed and interpreted the data and wrote the manuscript; W.R., M.Y., H.W., X.Y., D.L., and X.L. performed the bioinformatics analysis; X.W. and M.N. collected samples and clinical information; Y.H. and B.M. reviewed the pathological diagnosis; W.R., X.W., M.N., W.J., H.H., Z.L., and H.Z. interpreted the clinical information; D.L. and K.W. supervised the bioinformatics analysis; H.Z., K.W., and Z.L. were involved in study supervision; Q.P.-H. designed and supervised the study and revised the manuscript.

## Declaration of interests

The authors declare no competing interests.

## STAR★Methods

### Key resources table

**Table:** 

REAGENT or RESOURCE	SOURCE	IDENTIFIER
**Antibodies**		

Anti-CD4	Zsbio	Cat#: ZM-0418; RRID: AB_2890106
Anti-CD8	Roche	Cat#: 05937248001; RRID: AB_2335985

**Biological samples**		

Frozen tumor biopsies and FFPE sections	This paper	N/A
Peripheral blood DNA	This paper	N/A
Tumor biopsy RNA	This paper	N/A

**Critical commercial assays**		

DNeasy Tissue and Blood Kit	QIAGEN	Cat#: 69506
MGIEasy FS DNA Library Prep Set	MGI	Cat#: 1000006987
TruSeq DNA PCR-Free	Illumina	Cat#: FC-121-3003
TRIzol Reagent	Invitrogen	Cat#: 15596018
MGIEasy rRNA Depletion Kit	MGI	Cat#: 1000005953
Illumina TruSeq stranded mRNA	Illumina	Cat#: RS-122-2101

**Deposited data**		

Raw sequencing data	This paper	https://db.cngb.org, CNP0005740
Molecular subclusters of follicular lymphoma: a report from the United Kingdom’s Haematological Malignancy Research Network	Crouch et al.^33^	https://doi.org/10.1182/bloodadvances.2021005284
Identification of genetic subtypes in follicular lymphoma	Shelton et al.^34^	https://doi.org/10.1038/s41408-024-01111-w
Genetic subdivisions of follicular lymphoma defined by distinct coding and noncoding mutation patterns	Dreval et al.^35^	https://doi.org/10.1182/blood.2022018719

**Software and algorithms**		

Burrows-Wheeler Aligner (v0.7.15)	Li and Durbin^86^	RRID:SCR_010910; http://bio-bwa.sourceforge.net/
Picard (v2.18.7)	Broad Institute	RRID:SCR_006525; http://broadinstitute.github.io/picard/
Genome Analysis Toolkits (v4.1.0.0)	McKenna et al.^87^	RRID:SCR_001876; https://software.broadinstitute.org/gatk/
MuTect2 (v4.0.6.0)	Mckenna et al.^87^	RRID:SCR_001876; https://software.broadinstitute.org/gatk/
GISTIC (v2.0)	Mermel et al.^45^	RRID:SCR_000151; http://www.mmnt.net/db/0/0/ftp-genome.wi.mit.edu/distribution/GISTIC2.0
Limma	Ritchie et al.^88^	RRID:SCR_010943; https://bioconductor.org/packages/release/bioc/html/limma.html
GSEA	Subramanian et al.^89^	RRID:SCR_003199; http://software.broadinstitute.org/gsea/index.jsp
HISAT2 (2.1.0)	Kim et al.^90^	RRID:SCR_015530; http://ccb.jhu.edu/software/hisat2/index.shtml
SomaticSniper (v1.0.5.0)	Larson et al.^91^	RRID:SCR_005108; http://gmt.genome.wustl.edu/somatic-sniper/current/
Strelka2 (v2.9.2)	Kim et al.^92^	RRID:SCR_005109; https://github.com/Illumina/strelka
MuSE (v1.0)	Fan et al.^93^	RRID:SCR_026263; https://github.com/wwylab/MuSE
Svaba (v1.1.0)	Wala et al.^94^	RRID:SCR_022998; https://github.com/walaj/svaba
Manta (v1.6.0)	Chen et al.^47^	RRID:SCR_022997; https://github.com/Illumina/manta
Soapnuke (v2.1.5)	Chen et al.^95^	RRID:SCR_015025; https://github.com/BGI-flexlab/SOAPnuke
SigProfilerExtractor (1.1.23)	Bergstrom et al.^96^	RRID:SCR_023121; https://github.com/AlexandrovLab/SigProfilerExtractor/
FACETS (v0.15.0)	Shen et al.^46^	RRID:SCR_026264; https://github.com/mskcc/facets
Integrative Genomics Viewer (v2.8.2)	Robinson et al.^97^	RRID:SCR_011793; http://www.broadinstitute.org/igv/
NMF (v0.25)	Gaujoux et al.^98^	RRID:SCR_023124; https://cran.r-project.org/package=NMF
Liftover	Luu et al.^99^	RRID:SCR_018160; https://genome.ucsc.edu/cgi-bin/hgLiftOver
ActiveDriverWGS	Zhu et al.^100^	https://github.com/reimandlab/ActiveDriverWGSR
DriverPower (v1.0.2)	Shuai et al.^101^	https://github.com/smshuai/DriverPower
OncodriveFML (v2.4.0)	Mularoni et al.^102^	https://bbglab.irbbarcelona.org/oncodrivefml/home
xCell	Aran et al.^68^	RRID:SCR_026446; https://github.com/dviraran/xCell
Sklearn (v1.1)	Buitinck et al.^103^	RRID:SCR_019053; https://scikit-learn.org/stable/modules/generated/sklearn.decomposition.NMF.html
IntOGen (v1.1)	Martinez-Jimenez et al.^104^	https://www.intogen.org/search
SeekSV (v1.2.3)	Liang et al.^48^	https://github.com/qiukunlong/seeksv
Bam-matcher	Wang et al.^105^	https://github.com/edawson/bam-matcher
Lancet (v1.1.0)	Narzisi et al.^106^	https://github.com/nygenome/lancet
Mixcr (v3.0.13)	Bolotin et al.^107^	https://github.com/milaboratory/mixcr

### Experimental model and study participant details

A total of 131 FL patients, who were diagnosed at Tianjin Medical University Cancer Institute and Sun Yat-Sen University Cancer Center between 2006 and 2018 and whose frozen tumor tissues were available, were included in this study (Table S1). The pathological diagnosis of FL was made based on the WHO standard classification protocols (4th revised Edition and 5th Edition), and was confirmed by independent pathologists at the two cancer centers after molecular phenotyping. DNA samples from peripheral blood were available for 62 of these patients. The samples were obtained at the time of diagnosis or relapse prior to therapy. FL3B cases were included in our analysis, whereas duodenal-type and cutaneous follicle center lymphoma cases were excluded. Clinical data, including age, gender, Ann Arbor stage, pathology grade, performance status, IPI, and FLIPI, etc, were extracted from medical records. Those FL patients with chronic HBV infection did not suffer from other forms of immunodeficiency or chronic inflammation. Our FL patients received various treatments, including watchful waiting, radiotherapy, CHOP, and R-CHOP-like regimens. The clinical outcome and POD24 analyses were performed on those patients (*n* = 97) treated with R-CHOP-like regimens. The patients' response to the respective treatment was assessed using a modified version of the International Working Group response criteria. Patient characteristics, including age, gender, health status, treatment and other clinical factors are summarized in Tables S1 and S4, respectively. The informed consent was obtained from all patients, and ethical approval for the study was obtained from the Institutional Review Boards of Tianjin Medical University Cancer Institute, Sun Yat-Sen University Cancer Center and Hospital, and Karolinska Institutet.

### Method details

#### DNA extraction and WGS

DNA was extracted by using the DNeasy Tissue and Blood Kit (Qiagen, Venlo, Netherlands) following the manufacturer’s protocols. WGS was conducted on either the Illumina HiSeq 2000 (Illumina, San Diego, CA) or the DNBSEQ platform (MGI, Shenzhen, China). WGS was performed on 62 tumor samples from FL patients with paired control samples,^10^ while the remaining tumor samples were sequenced without paired controls. Detailed sequencing performance is presented in Table S1.

Alignment and quality control: Prior to read alignment, sequencing reads containing adapter sequences, low-quality reads (>10%), and low-quality bases (>50% bases with quality <5) were filtered out by using soapnuke (signature, soapnuke).^95^ The retained high-quality reads were then aligned to the human reference genome hg38 via Burrows–Wheeler Aligner software.^86^ Duplicate reads introduced by the polymerase chain reaction were identified and marked via MarkDuplicates (Picard, available at http://broadinstitute.github.io/picard/). Subsequent steps, including local realignment, quality score recalibration, and contamination analysis, were performed via the Genome Analysis Toolkit (GATK).^87^ To facilitate the comparison of Binary Alignment Map (BAM) files from matched tumor/control pairs, the BAM-matcher tool was utilized.^105^

Mutation calling: Somatic SNVs were detected via multiple tools, including MuSE, Strelka2, MuTect2, SomaticSniper, and Lancet, and insertion and deletion (InDel) variants were identified via Strelka2, MuTect2, Lancet, and Svaba with the default parameters and cutoffs.^87^^,^^91^^,^^92^^,^^93^^,^^94^^,^^106^ These tools were developed based on distinct algorithms, and their combination allows the complementary detection of somatic mutations. Somatic mutations identified by two or more pipelines were considered for subsequent analysis.

For tumor-only samples, to remove known germline events, SNVs and InDels with a variant allele frequency (VAF) ≥1% in gnomAD databases and germline events obtained from a panel of normal individuals were filtered out. The normal panel included the variants identified in the 62 control samples in our cohort, and germline events were identified via GATK’s HaplotypeCaller in at least two samples. Following filtering, a random forest method was employed to further identify somatic mutations in tumor-only samples.^108^ A total of 25 features were utilized to train the somatic mutation calling method in tumor (Table S6), which was implemented via the Python package 'sklearn'.^103^ The number of trees was set to 100. After mutation calling and/or filtering, we evaluated key parameters in both the paired and tumor-only sample cohorts, including overall sequencing depth, alternative (Alt) read depth, and VAF. Specifically, over 99.55% of the mutations had coverage of at least 10 reads, 99.87% were supported by at least 3 Alt reads, and 99.35% presented a VAF of ≥10%.

#### Mutational signature analysis

Mutational signatures were extracted from SNVs obtained from WGS data via SigProfiler (version 1.1.3).^96^ The suggested SBS96_*de novo* solution was then assigned to the COSMIC v3.2 reference (https://cancer.sanger.ac.uk/cosmic/signatures/SBS/) to determine the mutational processes that were active in our FL cohort.^109^ The process involved the following steps: (1) Somatic mutations from each dataset were categorized into 96 possible mutated trinucleotides, comprising 6 types of substitutions (C:G>A:T, C:G>G:C, C:G>T:A, T:A>A:T, T:A>C:G, and T:A>G:C) across 4 types of 5′ bases (A, C, G, T) and 4 types of 3′ bases (A, C, G, T), resulting in the creation of a mutational catalog. The frequency of each substitution type was subsequently computed for each individual sample. (2) The mutational signature framework was then utilized to decipher the signatures derived from the mutational catalog. (3) The number of extracted signatures (K) was set as previously described,^41^ and cosine similarity (θ) was used to estimate the similarity between signatures.

#### Detection of clustered somatic mutations (kataegis)

Kataegis regions were identified as previously described^40^^,^^41^ involving the following steps: (1) The abnormal distance line (ADL) was calculated, defined as one-tenth of the average distance between adjacent somatic mutations in each tumor sample. (2) The number of intermutation distances above and below the ADL was counted for every set of 10 adjacent mutations located within a 10 kb range. (3) A set of 10 adjacent mutations was identified as a kataegis if the fraction of intermutation distances below the ADL differed significantly from the overall distribution observed across all mutations in that sample (*p* < 0.0001, one-tailed Fisher’s exact test). (4) Overlapping kataegis regions were merged if the resulting *p* value for the merged region remained below 0.0001.

#### CNV analysis and estimation of tumor cell content

Somatic CNVs in both paired samples and tumor-only samples were analyzed via the CNV workflow in GATK.^87^ The workflow comprised two main components: (1) the denoising process, in which denoising was applied to the case sample alignment data against a panel of normal individuals (62 control samples from our cohort) to obtain copy ratios; and (2) segment modeling, in which the copy ratios and allelic counts were utilized to model segments. Additionally, we used FACETS^46^ to assess the accuracy of GATK by detecting CNVs in a subset of tumor samples. A comparison of the results from GATK and FACETS via IGV revealed highly similar results between the tools. Furthermore, we manually evaluated the alternative allele frequency of point mutations in each segment across all samples to ensure consistency in the observed changes. Significance analysis of recurrent broad and focal CNVs was conducted via the GISTIC2 algorithm,^45^ with the following parameters: -genegistic 1, -smallmem 0, -broad 1, -brlen 0.98, -armpeel 1, -savegene 1, - amplification 0.3, and - deletion 0.3. Regions with q values less than 0.25 are considered significant (default). To establish wide peak boundaries, a 90% confidence interval was specified. The tumor cell content was estimated from individual tumors via WGS data following the methodology described previously.^110^

#### Identification of structural variants (SVs)

Manta^47^ was utilized to detect SVs across the genome, whereas SeekSV^48^ was specifically employed to detect SVs within *IGH*. The results obtained from both tools were combined, and manual inspection was conducted via the Integrative Genomics Viewer (IGV).^97^ All identified SVs were supported by a minimum of three high-quality split reads (MQ ≥ 30). A review of the sequencing data in samples without *BCL2-IGH* SVs revealed several potential *BCL2-IGH* SVs supported by one or two high-quality split reads. For the seven *BCL2-IGH* SVs supported by two reads, breakpoint-specific PCR followed by Sanger sequencing was performed for validation. However, none of these translocations (supported by only two reads) were confirmed by PCR or Sanger sequencing. Additionally, the sequencing depth and coverage in the *BCL2* region were assessed in our FL samples.

#### Identification of significantly mutated genes (SMGs)

The IntOGen pipeline integrates outputs from seven different tools to compile a comprehensive list of candidate driver genes.^104^ These seven tools included dNdScv, OncodriveFML, OncodriveCLUSTL, cBaSE, Mutpanning, HotMaps3D, and smRegions. Each tool employs distinct features, such as linear clusters, 3D clusters, Pfam domains, excess mutations, and modes of action, to identify SMGs.^104^ During the integration process, IntOGen filtered out mutated genes related to signature 9 (COSMIC 9, associated with B-cell cancers). To recover these genes, we utilized the brown test provided by IntOGen. The criteria for inclusion were as follows: (1) brown test q < 0.05; (2) gene damage index (GDI) score<2000; (3) genes associated with important lymphoma-related functions; (4) mRNA of individual genes detected in FL tumors; and (5) mutation frequency >3% in our cohort.

#### Data combining from samples with and without matched controls

Using the 62 paired tumor/control samples as a benchmark, we compared the characteristics of SMGs, genome-wide mutational signatures, clustered mutation patterns (kataegis), and CNVs identified by both paired and tumor-only pipelines (Figure S8). These comprehensive analyses revealed minimal impact of residual germline variants on the identification of key somatic alterations (Figure S8). Therefore, data from the paired and tumor-only groups were merged for subsequent analyses. On average, we detected 2.8 mutations per megabase (Mb) in paired tumor/control samples and 6.0 mutations/Mb in tumor-only samples (Table S1). Within coding regions, paired samples had an average of 54 nonsilent mutations, compared to 142 in tumor-only samples. No significant associations between total mutation burden and key clinical parameters were observed in either group.

#### Nonnegative matrix factorization (NMF) consensus clustering

SMGs, significant regions of CNVs, *BCL2* SVs and *BCL6* SVs were assembled into a gene matrix (Table S3). These genetic lesions were assigned scores as follows: nonsilent mutations and indels, 2; synonymous mutations, 1; no mutation, 0; high-grade CNV gain (CNV ≥3.7 copies), 2; low-grade CNV gain (3.7 copies ≥ CNV ≥2.4 copies), 1; CNV-neutral, 0; low-grade CNV loss (0.80 ≤ CNV ≤1.6 copies), 1; high-grade CNV loss (CNV ≤0.80 copies), 2; and SV, 3. The NMF consensus clustering algorithm was applied to group samples into different clusters via NMF.^98^ Cophenetic coefficient values were calculated for K = 2 to K = 10 to determine the optimal solution, as depicted in Figures S2A and S2B (K = 3). Fisher’s exact test (*p* value ≤ 0.05) was used to identify markers associated with different clusters.^56^

#### Identification of noncoding drivers

Three approaches were employed to identify somatic noncoding drivers via SNVs, with reference noncoding genomic elements obtained from the Pan-cancer Analysis of Whole Genomes (PCAWG) study.^100^^,^^101^^,^^102^ Since the reference noncoding genomic elements are based on hg19, the SNV and Indel positions in hg38 were converted into the positions in hg19 via liftover tools prior to analysis.^99^

#### RNA extraction, transcriptome resequencing and gene set enrichment analysis (GSEA)

Transcriptome sequencing was conducted on 104 FL tumor samples, and total RNA was extracted via TRIzol reagent (Invitrogen, Paisley, UK). The sequencing libraries were prepared according to the manufacturer’s instructions and sequenced on the DNBSEQ platform or Illumina HiSeq2000 platform. The reads were aligned to the reference human genome and transcriptome hg38 via HISAT2.^90^ Gene expression levels were calculated via the number of transcripts per million (TPM) using RSEM.^111^ To remove batch effects, log2-transformed TPM values were normalized via the R package Limma.^88^ The normalized expression values were analyzed via Qlucore Omics Explorer (Qlucore AB, Lund, Sweden) or GSEA^89^ (Broad Institute, Cambridge, USA).

#### Prediction of tumor-infiltrating immune cells in FL tumors

Using RNA-seq data, the infiltration of various immune cell types was predicted using the online tool xCell (https://xcell.ucsf.edu/).^68^ Specifically, we employed the xCell algorithm, which leverages 1,822 human cell type transcriptomes and applies a curve-fitting method to compare cell types. Furthermore, we applied the default xCell signatures to RNA-seq data from our cohort to infer the abundance of various immune cell populations. The markers used for deconvolution are available in the original publication.^68^ An independent online cohort, GSE119214 (*n* = 137), consisting of FL patients treated with R-CHOP, was utilized for validation.^112^

#### Immunohistochemical (IHC) staining

IHC staining for CD4 (ZM-0418, Zsbio, China) and CD8 (Clone SP57, Roche, Switzerland), along with slide scanning, was performed in the Department of Pathology at Tianjin Medical University Cancer Institute. FFPE sections derived from 16 FL patients in our cohort were stained and analyzed. Expression levels were assessed via a semiquantitative scoring approach based on the staining intensity: 1 (<10%), 2 (10–20%), and 3 (>20%).

#### Characterization of *IGHV-IGHD-IGHJ-IGHC* in FLs tumors

The *IGHV-IGHD-IGHJ-IGHC* rearranged transcript sequences and BCR clonotype quantification were analyzed from RNA-seq data using MiXCR software,^107^ and the identification of tumor-derived rearranged transcripts followed the criteria previously described.^113^

### Quantification and statistical analysis

Quantification and statistical analyses were performed using R or GraphPad Prism 8. Categorical variables were compared using the chi-square test or Fisher’s exact test, as appropriate. For figures presenting pooled data with error bars, data are represented as mean ± SEM. The Mann–Whitney U test (two-tailed) was used for group comparisons. Progression-free survival (PFS) was defined as the time from diagnosis to disease recurrence, progression, death, or last follow-up, and overall survival (OS) as the time from diagnosis to death or last follow-up. Differences in PFS and OS between groups were assessed using the log rank test in Prism. For multiple comparisons, *p* values were adjusted using the Benjamini–Hochberg false discovery rate (FDR) correction and reported as q values. Correlations between continuous variables were evaluated using the Spearman correlation coefficient. Cosine similarity (θ) was used to estimate the similarity between signatures. Statistical significance was defined as *p* < 0.05.

## Supplemental information

Document S1. Figures S1–S8 and Tables S4–S6

Table S1. Clinical characteristics of patients with FL and sequencing performance of WGS in our cohort

Table S2. Comparison of gene mutation frequency among various cohorts

Table S3. Genetic features used for clustering analysis in our cohort

Document S2. Article plus supplemental information
