# Supplementary material for: Whole-genome sequencing reveals three follicular lymphoma subtypes with distinct cell of origin and patient outcomes
Source: Cell Rep Med. 2025 Aug 7;6(8):102278. doi: 10.1016/j.xcrm.2025.102278 (PMC12432372; doi:10.1016/j.xcrm.2025.102278)
Supplement: Document S1. Figures S1–S8 and Tables S4–S6 [file mmc1.pdf]

Cell Reports Medicine, Volume *6*

Supplemental information

Whole-genome sequencing reveals three follicular lymphoma subtypes with distinct cell of origin and patient outcomes

Weicheng Ren, Mingyu Yang, Xianhuo Wang, Man Nie, Yuhua Huang, Hui Wan, Dongbing Liu, Xiaobo Li, Xiaofei Ye, Bin Meng, Wenqi Jiang, Huiqiang Huang, Zhiming Li, Huilai Zhang, Kui Wu, and Qiang Pan-Hammarström

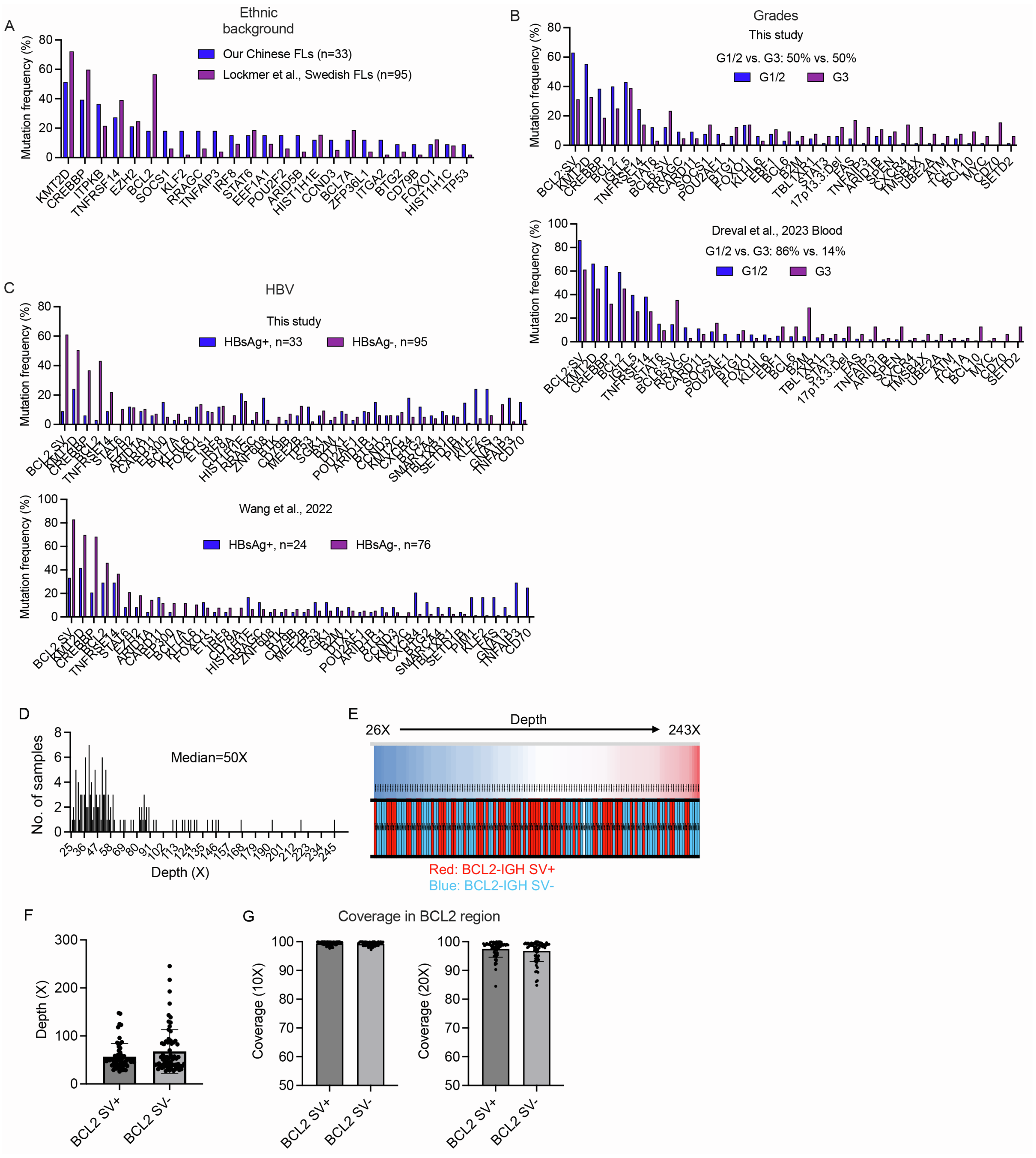

Figure S1. Comparison of gene mutation frequency in different groups derived from various studies and analysis of sequencing performance at the *BCL2* locus in our FL cohort, related to Figures 3 and 4.

(A) The FL tumors from our Chinese cohort and Swedish FL cohort from Lockmer et al.[44] were sequenced simultaneously using the same sequencing panel and analysis pipeline. (B-C) Mutation frequencies were compared across different FL grades (B) and HBV infection

statuses (C) using the data derived from various studies. G, grade. (D-G) The sequencing depth and coverage of the *BCL2* region were extracted and analyzed. (D) The distribution of sequencing depth. (E) Association between sequencing depth and *BCL2-IGH* translocation status. (F-G) Comparison of sequencing depth and coverage in the indicated groups. Panel D, F and G, data are represented as mean ± SEM. The Mann–Whitney U test was used to calculate the P value via the nonparametric test.

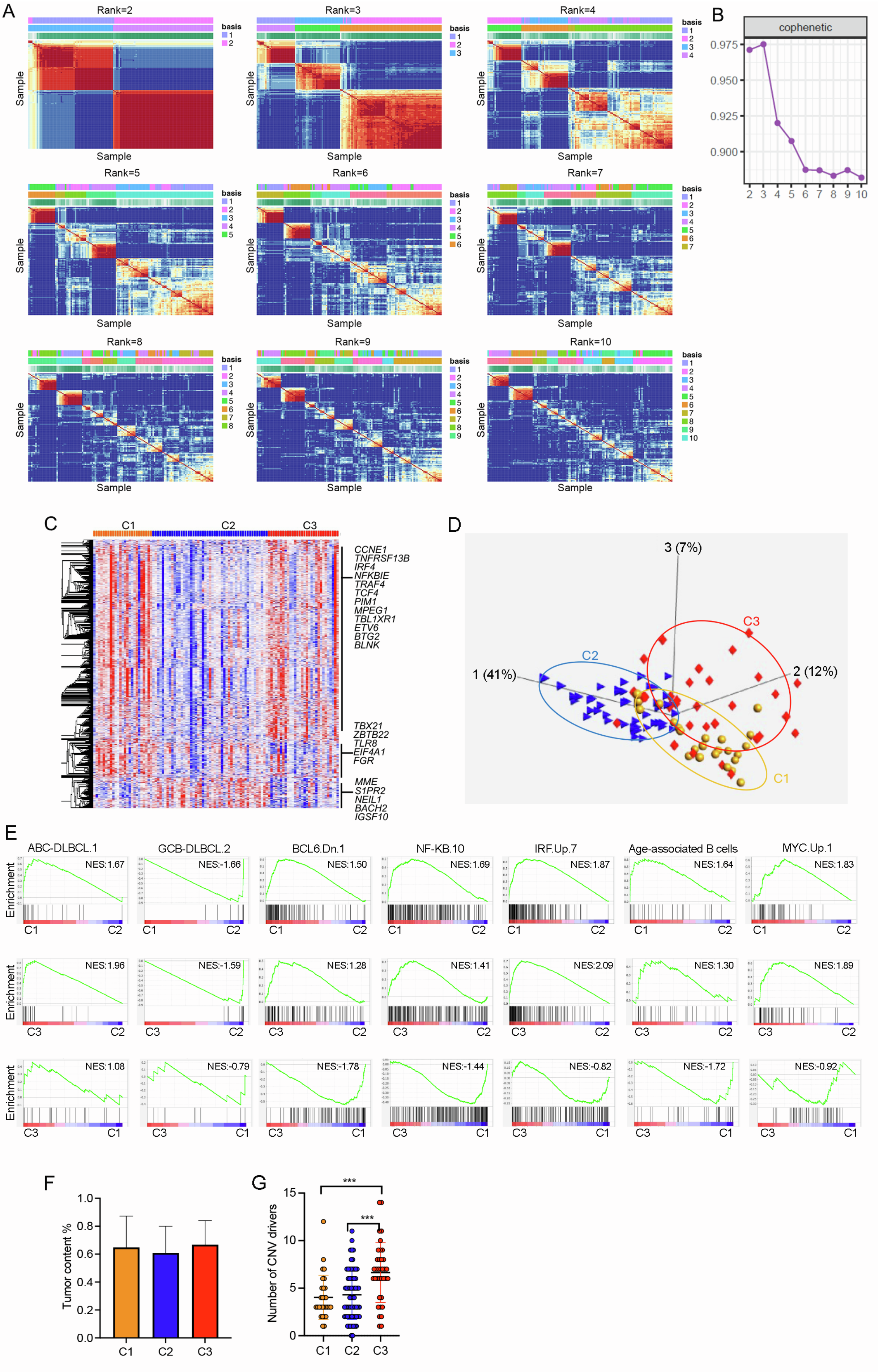
A
Rank=2
Rank=3
Rank=4
Rank=5
Rank=6
Rank=7
Rank=8
Rank=9
Rank=10
basis
Sample
B
cophenetic
0.975
0.950
0.925
0.900
2 3 4 5 6 7 8 9 10
C
C1
C2
C3
CCNE1
TNFRSF13B
IRF4
NFKBIE
TRAF4
TCF4
PIM1
MPEG1
TBL1XR1
ETV6
BTG2
BLNK
TBX21
ZBTB22
TLR8
EIF4A1
FGR
MME
S1PR2
NEIL1
BACH2
IGSF10
D
3 (7%)
1 (41%)
2 (12%)
C1
C2
C3
E
ABC-DLBCL.1
GCB-DLBCL.2
BCL6.Dn.1
NF-KB.10
IRF.Up.7
Age-associated B cells
MYC.Up.1
Enrichment
NES:1.67
NES:-1.66
NES:1.50
NES:1.69
NES:1.87
NES:1.64
NES:1.83
NES:1.96
NES:-1.59
NES:1.28
NES:1.41
NES:2.09
NES:1.30
NES:1.89
NES:1.08
NES:-0.79
NES:-1.78
NES:-1.44
NES:-0.82
NES:-1.72
NES:-0.92
F
Tumor content %
1.0
0.8
0.6
0.4
0.2
0.0
C1
C2
C3
G
Number of CNV drivers
15
10
5
0
***
***
C1
C2
C3

Figure S2. Consensus clustering of FL tumors and comparison of gene expression profiles, tumor cell content, and numbers of CNV drivers among FL genetic clusters, related to Figure 5.

(A) Consensus plots for cluster solutions ranging from k=2 to k=10 depict the degree of agreement among samples within each cluster for different values of k. (B) The cophenetic coefficient for cluster solutions ranging from k=2 to k=10 is calculated to assess the stability and quality of the clustering results. (C-E) The normalized expression levels were analyzed and compared. (C-D) Differentially expressed genes among the clusters were used to draw the heatmap (C) and PCA (D). (E) GSEA was performed to compare the enrichment of indicated genesets listed in the indicated clusters. (F-G) The tumor cell content (F) and the numbers of CNV drivers (G) were compared among the three FL genetic subtypes. Panel F and G, data are represented as mean ± SEM. The Mann–Whitney U test was used to calculate the P value. Statistical significance was defined as $p<0.05$. ***, $p<0.001$.

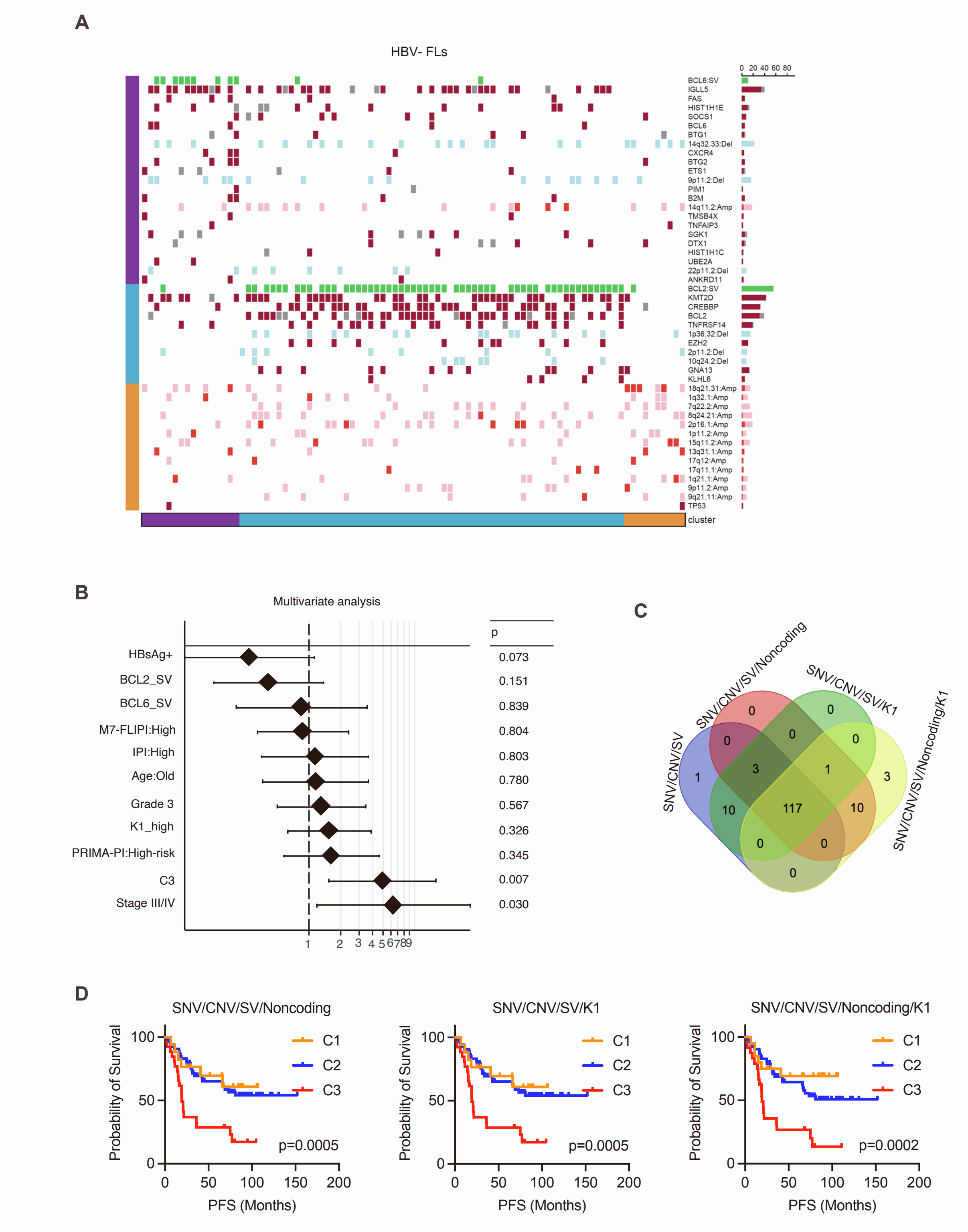

Figure S3. Genetic clustering in HBV- FL patients, multivariable Cox regression analyses and comparison of various genetic subtypes identified via different strategies, related to Figure 5.

(A) Genetic clusters in FL patients without HBV infection. The heatmap illustrates the mutational patterns observed in the three clusters of FL patients who were negative for the HBsAg serology test. (B) Multivariable Cox regression analyses demonstrating the independent prognostic value of the genetic clusters in our FL cohort, with the key parameters included in the analysis. Error bar represents the 95% confidence interval (lower to upper) of hazard ratio. P values were calculated using the Wald test. Statistical significance was defined as $p<0.05$. (C-D) Using the same clustering strategy, more types of genetic alterations, including kataegis and noncoding driver events, were tested via the subtyping approach. (C) The overlapping FL samples for the clusters identified by different strategies with various genetic alterations. (D) Kaplan–Meier survival analysis illustrating PFS among genetic subtypes identified from different strategies. In the PFS analysis, only patients treated with R-CHOP were included. The p value was calculated by the log-rank test.

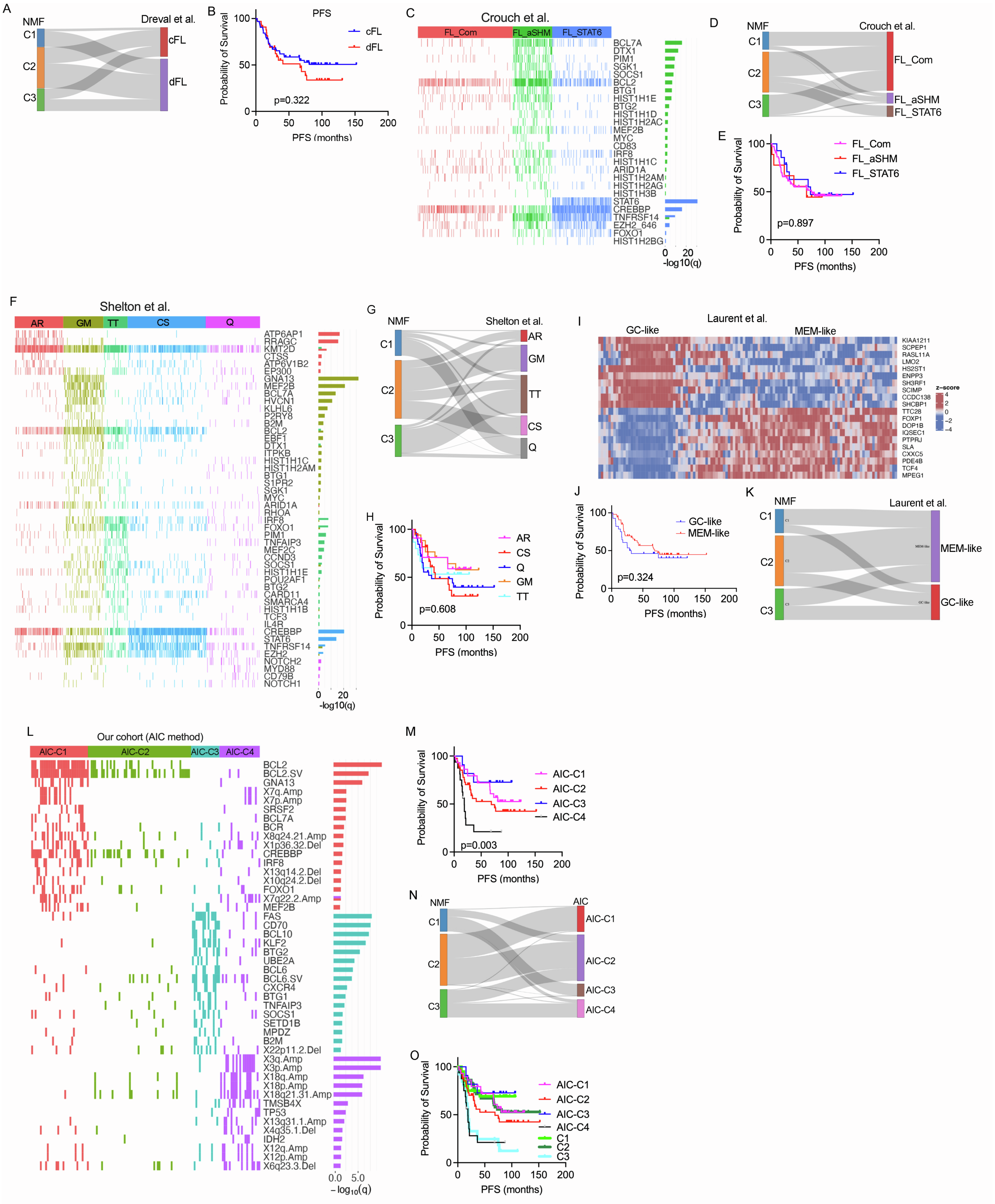

Figure S4. Alternative clustering solutions were performed in our FL cohort, related to Figure 5.

Clustering analyses were conducted using the genetic markers and methods described in the referenced studies Dreval et al., Crouch et al., Shelton et al., Laurent et al.[32-35]. Our samples

were merged with their FL samples for clustering, and subsequent PFS analysis was performed within our cohort. (A) The correlation between different approaches and (B) PFS analysis of clusters within our cohort. The data from Table S4 (Dreval et al.) was used to calculate the median mutation numbers for each aSHM region across all DLBCLs (n=195). In our cohort, FL samples were considered positive for aSHM if the number of mutations in each aSHM region exceeded the median by at least 3, consistent with the criteria established by Dreval et al. (2023). In the prediction pipeline, the suggested cutoff of 0.8 (FL.prob.) was applied to predict cFL and dFL in our cohort. (C) Clusters identified using markers from Crouch et al. Sample number: n=844. (D) Sankey plot showing the correlation between different approaches. (E) PFS analysis of clusters within our cohort. (F) Clusters identified using markers from Shelton et al. Sample number: n=679. (G) Sankey plot showing the correlation between different approaches. (H) PFS analysis of clusters within our cohort. In the PFS analysis, only patients treated with R-CHOP were included. (I) Clusters identified using markers from Laurent et al., using RNAseq data (J) PFS analysis of clusters within our cohort. (K) Sankey plot showing the correlation between indicated approaches. (L-O) The previously described Bernoulli mixture model was applied to our FL dataset using our genetic markers, including SNVs, CNVs, and SVs. Results include: (L) mutation patterns across the identified clusters. (M) PFS analysis for these clusters, and (N-O) Comparisons between clusters identified using different clustering approaches. In the PFS analysis, only patients treated with R-CHOP were included. Panel B, E, H, M and O, the p value was calculated by the log-rank test. AIC, Akaike Information Criterion. NMF, Non-negative matrix factorization.

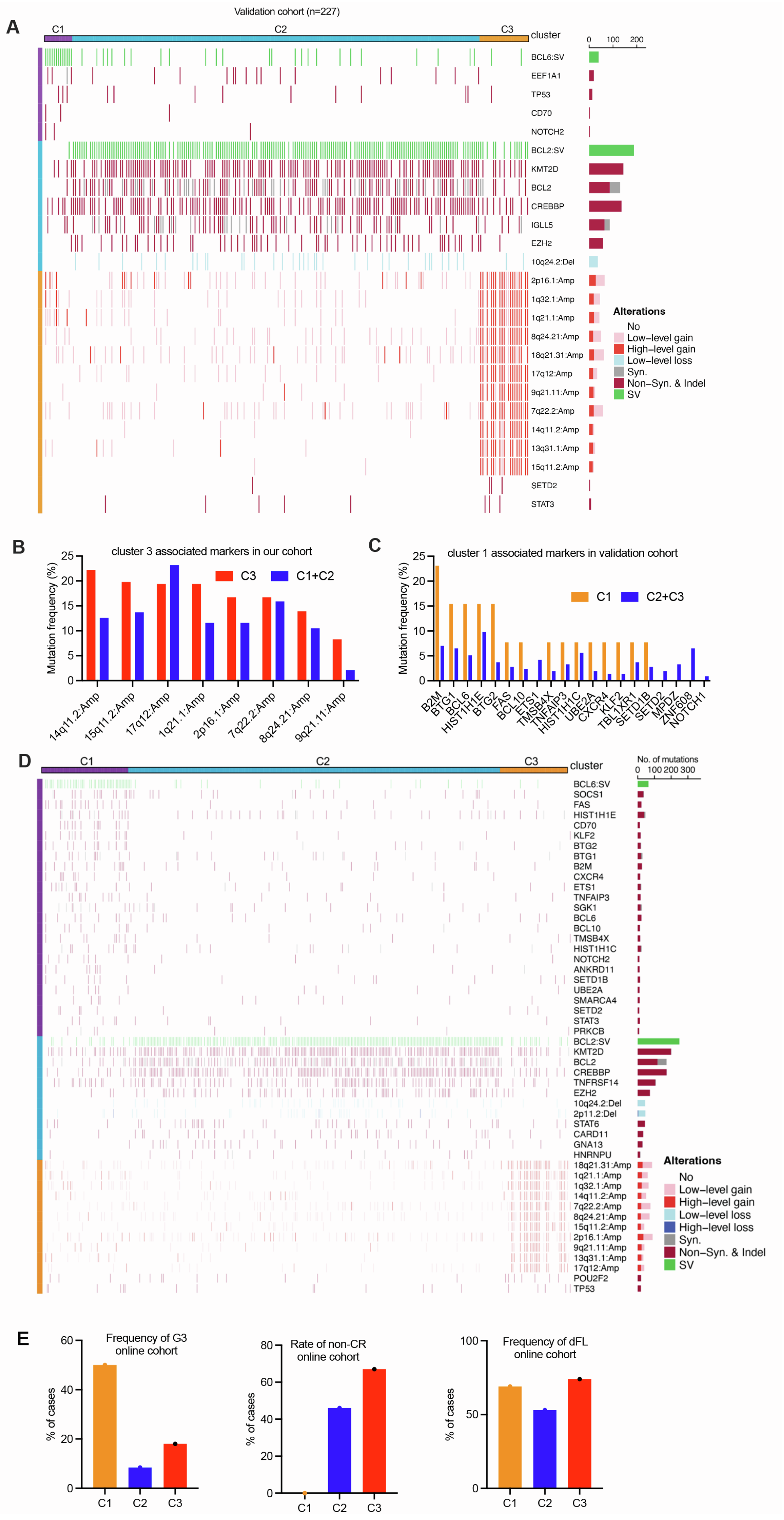

A
Validation cohort (n=227)
C1
C2
C3
cluster
0 100 200
BCL6:SV
EEF1A1
TP53
CD70
NOTCH2
BCL2:SV
KMT2D
BCL2
CREBBP
IGLL5
EZH2
10q24.2:Del
2p16.1:Amp
1q32.1:Amp
1q21.1:Amp
8q24.21:Amp
18q21.31:Amp
17q12:Amp
9q21.11:Amp
7q22.2:Amp
14q11.2:Amp
13q31.1:Amp
15q11.2:Amp
SETD2
STAT3
Alterations
No
Low−level gain
High−level gain
Low−level loss
Syn.
Non−Syn. & Indel
SV
B
cluster 3 associated markers in our cohort
Mutation frequency (%)
C3
C1+C2
14q11.2:Amp
15q11.2:Amp
17q12:Amp
1q21.1:Amp
2p16.1:Amp
7q22.2:Amp
8q24.21:Amp
9q21.11:Amp
C
cluster 1 associated markers in validation cohort
Mutation frequency (%)
C1
C2+C3
B2M
BTG1
BCL6
HIST1H1E
BTG2
FAS
BCL10
ETS1
TMSB4X
TNFAIP3
HIST1H1C
UBE2A
CXCR4
KLF2
TBL1XR1
SETD1B
SETD2
MPDZ
ZNF608
NOTCH1
D
C1
C2
C3
cluster
No. of mutations
0 100 200 300
BCL6:SV
SOCS1
FAS
HIST1H1E
CD70
KLF2
BTG2
BTG1
B2M
CXCR4
ETS1
TNFAIP3
SGK1
BCL6
BCL10
TMSB4X
HIST1H1C
NOTCH2
ANKRD11
SETD1B
UBE2A
SMARCA4
SETD2
STAT3
PRKCB
BCL2:SV
KMT2D
BCL2
CREBBP
TNFRSF14
EZH2
10q24.2:Del
2p11.2:Del
STAT6
CARD11
GNA13
HNRNPU
18q21.31:Amp
1q21.1:Amp
1q32.1:Amp
14q11.2:Amp
7q22.2:Amp
8q24.21:Amp
15q11.2:Amp
2p16.1:Amp
9q21.11:Amp
13q31.1:Amp
17q12:Amp
POU2F2
TP53
Alterations
No
Low−level gain
High−level gain
Low−level loss
High−level loss
Syn.
Non−Syn. & Indel
SV
E
Frequency of G3
online cohort
% of cases
Rate of non-CR
online cohort
Frequency of dFL
online cohort
C1
C2
C3

Figure S5. Identification of FL subtypes in an independent validation cohort, related to Figure 5.

Using the same genetic alterations identified in our cohort, we applied the same clustering strategy to a validation cohort comprising 227 samples from Dreval et al.[35]. All these genetic changes were obtained from the paper directly. (A) Three clusters were distinguished by distinct genetic alterations. (B) Comparison of the mutation frequencies of C3-associated CNV markers in our cohort. (C) Comparison of the mutation frequency of C1-associated marker genes in the validation cohort. (D) Analysis of FL subtypes in a combined FL cohort (n=358), including our cohort (n=131) and validation cohort (n=227). A nonnegative matrix factorization consensus clustering approach combining SMGs, CNVs, and SVs was used to classify FL tumors. (E) Analysis of FL grades and treatment outcomes across the three clusters in the validation cohort. Comparison of treatment outcomes was performed only for patients treated with R-CHOP. CR, complete remission. Panel A and D, $P<0.05$ was used to define the markers of individual clusters. Fisher's exact test was used to calculate the p value.

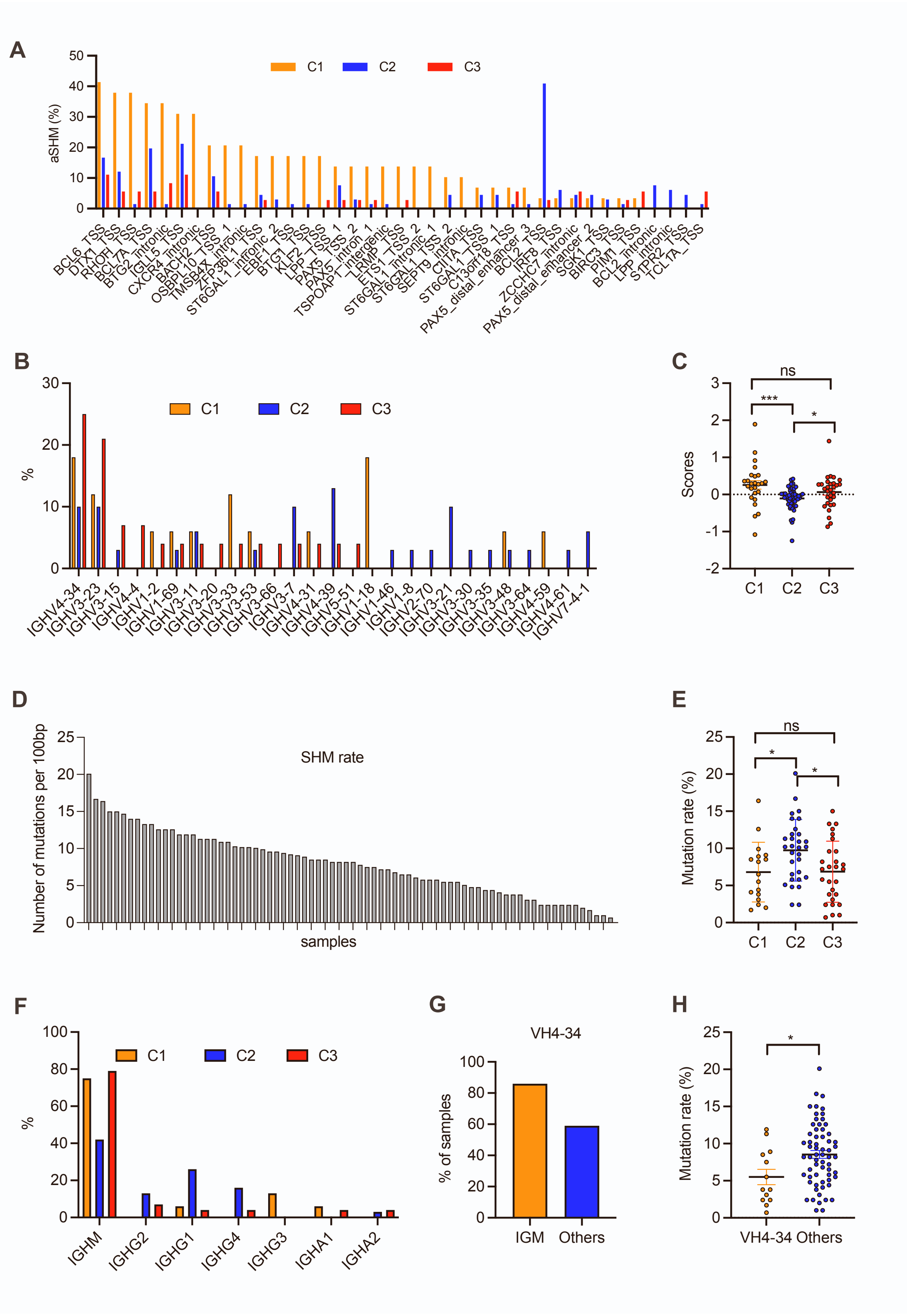

Figure S6. Comparison of the frequency of aberrant somatic hypermutation (aSHM) regions and IGH repertoires among different FL genetic subtypes, related to Figure 6.

(A) The 88 aSHM regions identified in Dreval et al.[35] were extracted and compared across the three genetic clusters identified in our cohort. The data from Table S4 (Dreval et al.) was used to calculate the median number of mutations for each aSHM region across all DLBCLs in their cohort (n=195). In our cohort, samples were considered positive for aSHM if the number of mutations in each aSHM region exceeded the median by at least 3, consistent with the criteria established by Dreval et al. Comparisons were made only for the aSHM regions identified in at least 3 FL samples from our cohort (n=39). Freq., frequency. TSS, translation start site. (B-H) Diverse IGH repertoires among different FL genetic subtypes. The *IGHV* sequences of the major tumor clones were analyzed based on RNAseq data. (B) The composition of the *IGHV* gene usage across the three genetic clusters. (C) The expression scores of systemic lupus erythematosus-associated pathway in the KEGG database. (D) The SHM rate identified across all FL tumors. (E) Comparison of the SHM rates in the three clusters. (F) Comparison of the frequency of Ig isotypes expressed on the major tumor clones in the samples belong to the three clusters. (G) Pairing of *VH4-34* with IGM and other isotypes. (H) Comparison of the SHM rates of *VH4-34* and other *VH* genes. Panel C, E and H, data are represented as mean ± SEM. The Mann–Whitney U test was used to calculate the P value via the nonparametric test. Statistical significance was defined as $p<0.05$. *, $p<0.05$. ***, $p<0.001$. ns, not significant.

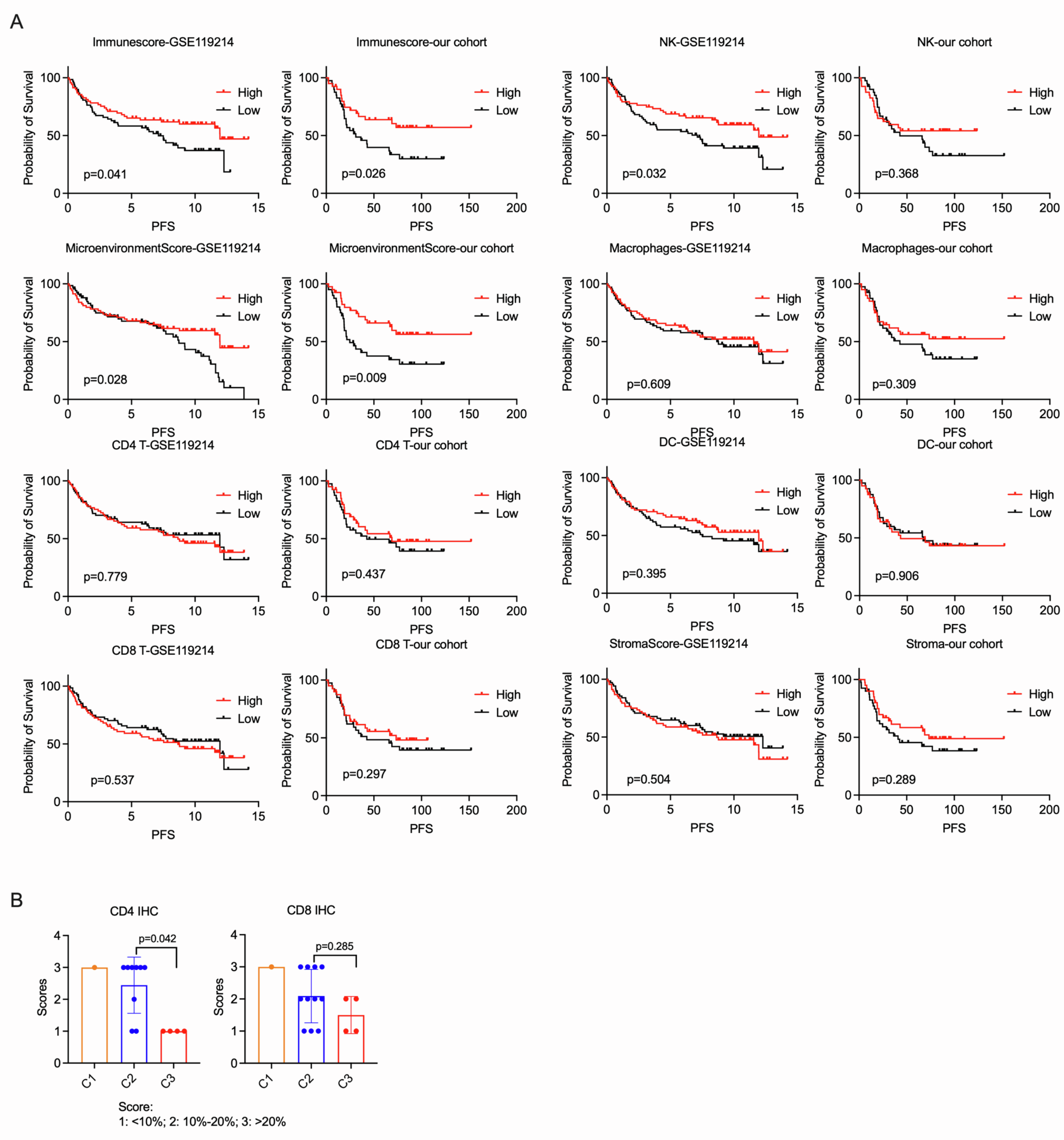

Figure S7. Analysis of the association between tumor-infiltrating immune cell scores and patient outcomes and CD4 and CD8 protein expression in FL tumors, related to Figure 6. (A) Gene expression data were used to predict the abundance of tumor-infiltrating immune cells using the online tool xCell in our cohort and the GSE119214 cohort (n=137), respectively. Kaplan–Meier survival analysis was used to illustrate PFS in the indicated groups among patients treated with R-CHOP. The median value of the individual scores was used to assign

samples into high and low groups. The p value was calculated by the log-rank test. (B) Immunohistochemistry (IHC) staining for CD4 and CD8 was performed on a subset of available samples (n=16). We quantified CD4 and CD8 expression levels in individual tumor samples using a semi-quantitative scoring method: 1: <10%, 2: 10–20%, and 3: >20%, respectively. Data are represented as mean ± SEM. The Mann–Whitney U test was used to calculate the P value via the nonparametric test.

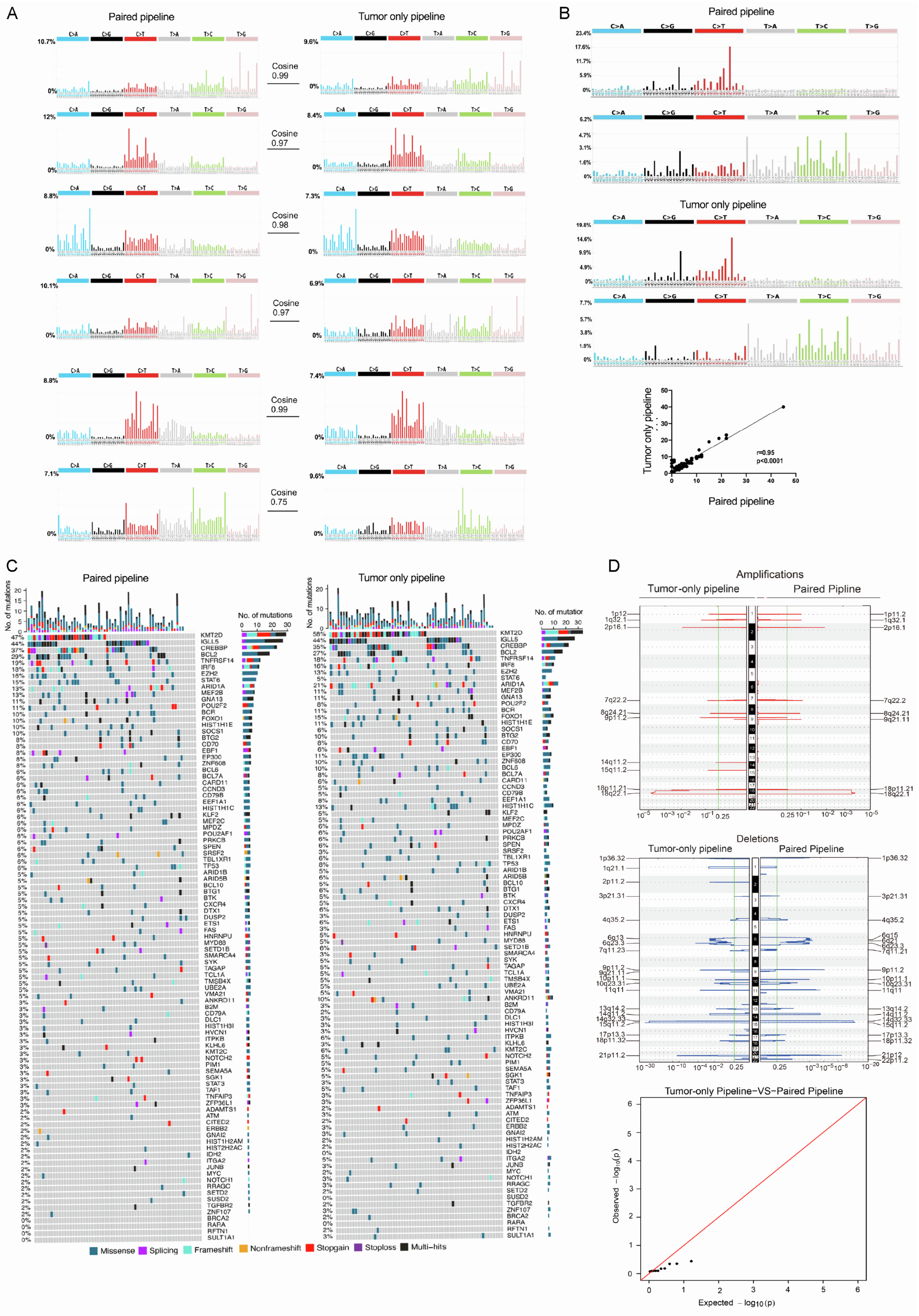
A
Paired pipeline
Tumor only pipeline
Cosine 0.99
Cosine 0.97
Cosine 0.98
Cosine 0.97
Cosine 0.99
Cosine 0.75
B
Paired pipeline
Tumor only pipeline
r=0.95
p<0.0001
Paired pipeline
C
Paired pipeline
Tumor only pipeline
No. of mutations
Missense
Splicing
Frameshift
Nonframeshift
Stopgain
Stoploss
Multi-hits
D
Amplifications
Tumor-only pipeline
Paired Pipline
Deletions
Tumor-only pipeline
Paired Pipeline
Tumor-only Pipeline-VS-Paired Pipeline
Observed −log10(p)
Expected −log10(p)

Figure S8. Comparison of somatic alterations identified in paired tumor/control pipeline and tumor-only pipeline, related to Figures 1-3.

The 62 paired samples were used for this comparison. Mutations identified by paired tumor/control pipeline and tumor-only pipeline were analyzed separately. (A) Mutational signatures were analyzed separately using mutation data derived from either paired tumor/control pipeline or tumor-only pipeline. (B) Kataegis was identified in individual samples, and subsequently, the mutational signatures of kataegis were identified separately for tumor DNA derived from paired and tumor-only samples. The image shows a correlation comparing the number of kataegis events identified using SNVs called by different pipelines. (C) Comparison of significantly mutated genes called using paired tumor/control pipeline and tumor-only pipeline, respectively. (D) The 62 paired samples were used for this comparison, and GATK was used to detect CNVs using either paired tumor/control setting or tumor-only setting. Subsequently, GISTIC2 was employed to detect focal CNVs separately. The CNV amplifications and deletions identified from different analysis pipelines were compared and correlated. Each plot represents one focal CNV. Panel B and D, the Spearman correlation was used to calculate the r and p values.

Table S4. Clinical characteristics of different FL genetic subtypes in all samples, related to Figure 5.

	C1	C2	C3
Age (years)	29	66	36
> 60	9 (31%)	20 (30%)	6 (17%)
≤ 60	20 (69%)	46 (70%)	30 (83%)
Gender			
Female	15 (52%)	27 (42%)	13 (36%)
Male	14 (48%)	39 (58%)	23 (64%)
Elevated LDH			
Yes	5 (19%)	10 (16%)	15 (44%)
No	21 (81%)	52 (84%)	19 (56%)
Stage			
I-II	8 (31%)	11 (17%)	4 (12%)
III-IV	18 (69%)	53 (83%)	29 (88%)
IPI			
0-2	20 (77%)	50 (78%)	27 (77%)
3-5	6 (23%)	14 (22%)	8 (23%)
Grade			
1-2	10 (34%)	43 (65%)	13 (36%)
3	19 (76%)	23 (35%)	23 (64%)
HBsAg			
Yes	15 (56%)	6 (11%)	12 (48%)
No	12 (44%)	60 (89%)	23 (52%)
CD10 IHC			
Positive	20 (71%)	65 (98%)	25 (71%)
Negative	8 (29%)	1 (2%)	10 (29%)
BCL6 IHC			
Positive	25 (89%)	60 (92%)	34 (97%)
Negative	3 (11%)	5 (8%)	1 (3%)
BCL2 IHC			
Positive	26 (93%)	65 (98%)	35 (100%)
Negative	2 (7%)	1 (2%)	0 (0%)
CD23 IHC			
Positive	15 (79%)	51 (93%)	26 (90%)
Negative	4 (21%)	4 (7%)	3 (10%)
MUM1 IHC			
Positive	4 (36%)	7 (20%)	13 (52%)
Negative	7 (64%)	28 (80%)	12 (48%)
POD24 (R-CHOP)			
Yes	5 (25%)	7 (14%)	17 (68%)
No	15 (75%)	43 (86%)	8 (32%)
POD24 (CHOP and others)			
Yes	4 (100%)	3 (50%)	3 (75%)
No	0 (0%)	3 (50%)	1 (25%)
FLIPI score			
0-1	5 (20%)	10 (17%)	4 (13%)
2	10 (40%)	15 (25%)	7 (23%)
3-5	10 (40%)	34 (58%)	19 (66%)
M7-FLIPI			
High-risk	4 (17%)	16 (27%)	7 (23%)
Low-risk	20 (83%)	43 (73%)	23 (77%)
PRIMA-PI			
High-risk	6 (43%)	12 (27%)	10 (36%)
Low/int-risk	8 (57%)	33 (73%)	18 (64%)

The numbers represent the numbers of samples with available data. The values are presented as n (%).

Table S5. Clinical characteristics of different genetic clusters in HBsAg- FL patients, related to Figure 5.

	C1	C2	C3
Age (years)	12	60	23
> 60	6 (50%)	19 (32%)	4 (17%)
≤ 60	6 (50%)	41 (68%)	19 (83%)
Gender			
Female	7 (58%)	25 (42%)	7 (30%)
Male	5 (42%)	35 (58%)	16 (70%)
Elevated LDH			
Yes	2 (18%)	9 (16%)	8 (36%)
No	9 (82%)	48 (84%)	14 (64%)
Stage			
I-II	6 (55%)	10 (17%)	4 (19%)
III-IV	5 (45%)	48 (83%)	17 (81%)
IPI			
0-2	9 (75%)	46 (78%)	19 (83%)
3-5	3 (25%)	13 (22%)	4 (17%)
Grade			
1-2	7 (58%)	40 (67%)	12 (52%)
3	5 (42%)	20 (33%)	11 (48%)
POD24 (R-CHOP)			
Yes	1 (11%)	5 (11%)	10 (67%)
No	8 (89%)	42 (89%)	5 (33%)
POD24 (CHOP and others)			
Yes	1 (50%)	3 (27%)	2 (50%)
No	1 (50%)	8 (73%)	2 (50%)
FLIPI score			
0-1	2 (22%)	10 (19%)	3 (14%)
2	5 (56%)	12 (23%)	7 (33%)
3-5	2 (22%)	32 (59%)	11 (53%)
M7-FLIPI			
High-risk	2 (22%)	15 (28%)	3 (14%)
Low-risk	7 (88%)	39 (72%)	18 (86%)
PRIMA-PI			
High-risk	3 (42%)	12 (28%)	8 (40%)
Low/int-risk	4 (58%)	31 (72%)	12 (60%)

The numbers represent the numbers of samples with available data. The values are presented as n (%).

Table S6. Features used to train the method of calling mutations in tumor-only samples, related to Figures 1-3.

VAF	Variant allele frequency, which is the propotion of reads supporting variant allele.
sample_freq	The proportion of mutations occurring in all samples
dbsnp	If the variation site is located in SNP database,the value is 1; otherwise the value is 0.
ref_depth	log(1+n), where n is the number of reads that support the reference allele.
Ref_End[1]	log(1+n), where n is the number of reads that support the reference allele and the base is located in the end regions of those reads.
ref_mid[2]	log(1+n), where n is the number of reads which support the reference allele and the base is located in middle of those reads.
ref_minus[3]	log(1+n), where n is the number of reads which support the reference allele and those reads are from minus.
Ref_Plus[4]	log(1+n), where n is the number of reads which support the reference allele and those reads are from plus.
alt_depth	log(1+n), where n is the number of reads that support the variant allele.
alt_plus	log(1+n), where n is the number of reads which support the variant allele and those reads are from plus.
alt_minus	log(1+n), where n is the number of reads which support the variant allele and those reads are from minus.
alt_mid	log(1+n), where n is the number of reads which support the reference allele and the base is located in middle of those reads.
Alt_End	log(1+n), where n is the number of reads that support the variant allele and the base is located in the end regions of those reads.
Ti/Tv	Transition-to-transversion ratio
strandbias_p	The p value of fisher exactly test according to whether the reads support minus or plus and the reads support variation or reference.
Strandbias_ratio[5]	The formula is as below.
sample_depth	log(1+n), where n is the average depth of each sample
depth_site	log(1+n), where n is the number of reads which support the variant and reference allele.
sequence_context	96 different contexts, considering not only the mutated base, but also the bases immediately 5' and 3'.
substitution_pattern	If the mutation type is A to T or T to A, the value is 1; If the mutation type is A to C or T to G, the value is 2; If the mutation type is A to G or T to C, the value is 3; If the mutation type is G to C or C to G, the value is 4; If the mutation type is G to A or C to T, the value is 5; If the mutation type is G to T or C to A, the value is 6;
End_ratio[6]	The formula is as below.
End_p	The p value of fisher exactly test according to whether the base is located in the end of reads and the reads support variation or reference.
simplerepeat	If the variation site located in simple repeat database, the value is 1; otherwise the value is 0.
cosmic	If the variation site is located in COSMIC database,the value is 1; otherwise the value is 0.
Bino	If the variation site has passed the binomial test, the value is 1; otherwise it is 0.

[1] End：The base is located in the region of 5bp 5'reads or 10bp 3' reads.

[2] Mid：The base is not located in end of reads region.

[3] Plus: The reads mapping to the forward strand.

[4] Minus: The reads mapping to the reverse strand.

[5]If Alt_Reads(mid) or Ref_Reads(mid) or (Ref_Reads(end)/Ref_Reads(mid) equal 0, the value of End_ratio is 10. if End_ratio is larger 10, the End_ratio is also is 10.

$$\mathrm{End}_{\mathrm{ratio}} = \frac{\mathrm{Alt\_Reads(end)/Alt\_Reads(mid)}}{\mathrm{Ref\_Reads(end)/Ref\_Reads(mid)}}$$

[6]If Alt_Reads(plus) or Ref_Reads(plus) or (Ref_Reads(minus)/Ref_Reads(plus) equal 0, the value of End_ratio is 10. if Strandbias_ratio is larger 10, the Strandbias_ratio is also is 10.

$$\mathrm{Strandbias}_{\mathrm{ratio}} = \frac{\mathrm{Alt\_Reads(minus)/Alt\_Reads(plus)}}{\mathrm{Ref\_Reads(minus)/Ref\_Reads(plus)}}$$
